# Hospitalisations and deaths due to ambulatory care sensitive conditions among adults with and without intellectual disabilities in Scotland: a cohort study

**DOI:** 10.1136/bmjopen-2025-105055

**Published:** 2026-03-02

**Authors:** Filip Sosenko, Deborah Cairns, Bhautesh Jani, Laura McKernan Ward, Maria Truesdale, Laura Hughes-McCormack, Angela Henderson, Craig Melville

**Affiliations:** 1 School of Health and Wellbeing, University of Glasgow College of Medical Veterinary and Life Sciences, Glasgow, UK; 2 School of Health and Wellbeing, University of Glasgow, Glasgow, UK; 3 University of Glasgow, Glasgow, UK; 4 Health Informatics Centre, University of Dundee School of Medicine, Dundee, UK; 5 Mental Health and Wellbeing, University of Glasgow School of Health and Wellbeing, Glasgow, UK; 6 Mental Health and Wellbeing, University of Glasgow College of Medical Veterinary and Life Sciences, Glasgow, UK

**Keywords:** Primary Care, Mortality, Hospitalization

## Abstract

**Abstract:**

**Objectives:**

To explore how well the primary care system in Scotland works for adults with intellectual disabilities (ID), using the rate of unplanned hospital admissions for ambulatory care sensitive conditions (ACSC) as a proxy indicator. As part of this, to investigate those rates and rate ratios among adults with ID and without ID, adjusting for the prevalence of a given ACSC in each population. The secondary aim was to explore deaths due to ACSC among the ID and no-ID populations.

**Design:**

A population-based retrospective cohort data linkage study of adult respondents to Scotland’s 2011 Census. Self-reported or proxy-reported ID status from the Census was linked to hospital admissions data and deaths data. The cohort was followed until the end of 2019. The prevalence of ACSCs in each population was calculated from aggregate-level data published by the National Health Service, as it was not possible to use the linked dataset for this purpose.

**Setting:**

Whole population of Scotland.

**Participants:**

People aged 18+ on census day (27 March 2011), including all adults with ID (n=16 840) and a 15% randomly selected comparator sample of adults without ID (n=566 074).

**Primary and secondary outcome measures:**

Crude and age-sex standardised incidence rates and ratios; cumulative incidence; prevalence ratios. The exposure was ID status, and the outcomes were (1) unplanned ACSC hospital admission, (2) death with an ACSC condition listed as the main cause on the death certificate and (3) death with an ACSC condition listed as one of the causes on the death certificate.

**Results:**

Adults with ID under the age of 55 had only a slightly higher risk of an unplanned ACSC hospitalisation than their general population counterparts (standardised incidence ratio 1.11; 95% CI 1.03 to 1.20). After adjusting for different ACSC prevalence in ID and non-ID cohorts, this difference in risk disappeared. These findings contrast with existing evidence from England, where a much higher unadjusted risk of unplanned ACSC hospitalisations was found among people with ID. Adults with ID had a higher risk of dying due to ACSC than adults without ID (standardised mortality ratio 2.54; 95% CI 2.19 to 2.95).

**Conclusions:**

Our findings on unplanned ACSC hospitalisations suggest that the primary care system in Scotland appears to be similarly effective for adults with ID than for adults without ID. However, the higher risk of dying from ACSC among people with ID suggests that this system is less effective for people with ID. Future research should investigate this tension and aim to understand why the operation of the primary healthcare system seems to be worse with regards to ACSC mortality than with regards to unplanned ACSC hospitalisations.

**STRENGTHS AND LIMITATIONS OF THIS STUDY**

- The study used data of highest available quality and largest available coverage.
- The study took into account different ambulatory care sensitive conditions (ACSCs) prevalence among people with and without intellectual disabilities (ID).
- The study investigated deaths due to ACSC, an under-researched area.
- Small absolute size of the ID population in Scotland entailed that CIs were wide for most ACSCs.
- It was impossible to carry out a sensitivity analysis, as Scotland does not have an intellectual disability register and researchers do not have access to patient-level primary care records.

## Introduction

 Adults with intellectual disabilities (ID) often experience complex and multiple health needs^1^,^3^ which are different to those experienced by adults in the general population.^2^ Frequency of hospital admissions for adults with ID has also been reported as higher^4 5^ and evidence suggests that adults with ID face numerous barriers to managing long-term conditions in primary care due to: communication difficulties; partial health literacy; difficulty understanding health information; and patients feeling fear or embarrassment.^6 7^ Barriers are also created through lack of education/knowledge about people with ID from healthcare professionals; inadequate coordination and continuity of care; and limited involvement of people with ID and supporters/carers in healthcare decision-making.^68^,^10^ Due to the existence of these barriers, annual health checks and other such active interventions are being introduced for adults with ID.^11^

Since some disadvantaged groups in the population, including people with ID, have more limited access to/engagement with primary care, or may receive less effective care, some researchers have argued that the rate of unplanned hospital admissions for ambulatory care sensitive conditions (ACSC) (such as asthma or diabetes) can be used as a proxy indicator of how well the primary care system works for a population of interest.^12^,^19^ Thus, a higher rate of unplanned hospital admissions for ACSC is taken as an indicator of a less effective primary care system.

Several empirical studies, particularly in the US, have shown that in countries where access to primary healthcare is not universal, people experiencing socio-economic disadvantage (who thus have more limited access) have a higher rate of unplanned hospitalisations for ACSC.^20^,^23^ However, some studies have not found any such link.^22 24 25^ A counter-argument has also been raised that it is not obvious that unplanned hospitalisations that occur are truly preventable.^26^ It has also been pointed out that primary care providers should not be held responsible for unplanned hospitalisations where the subjects did not seek medical care.^22^

With regard to substantive findings about ACSC among people with ID, previous research in England found that such people were consistently hospitalised at a higher rate for ACSC than people without an ID.^19 27 28^ However, neither of these studies controlled for the prevalence of ACSC in the analyses. A US study on diabetes and asthma did control for different prevalences and reported that the hospitalisation rates for diabetes and asthma were higher for adults with ID.^29^ Another US study found that ACSC hospitalisation rates were higher for women than men with ID.^30^ A study in England found that health checks in primary care reduced emergency admissions for ACSC,^31^ while a US study has found that people residing at home with no health support services had a higher emergency hospital visit rate than people with other residential circumstances.^32^

In this paper, we take a view that unplanned hospitalisation rates for ACSC are a useful indicator of the extent to which there is a ‘problem’ when it comes to access to primary care, or the quality of care received, for a specific group cf. the general population. We do agree, however, with an argument made by some researchers (eg, a study by Eggli *et al*^22^) that the rate needs to be adjusted for the prevalence of a given condition in each population being compared. It is logical to expect more ACSC hospitalisations in the population with a higher prevalence of ACSC. However, as all proxy measures, unplanned hospitalisation rates for ACSC lack nuance as an indicator of the quality of primary care services. There is also an additional element when considering the effectiveness of primary care for adults with ID because the initial presentation to primary care services is often dependent on family or paid carers recognising there is a problem. Nonetheless, our view is that unplanned ACSC hospitalisation rate gives an overall indication of the effectiveness of the reactive primary care system for adults with ID.^33^

The main aim of this study was to investigate whether the unplanned ACSC hospitalisation rate among people with ID in Scotland is higher than the general population rate, adjusting for differences in the prevalence of ACSCs. An additional aim was to compare mortality due to ACSC, a topic that has received relatively less attention so far.^18 34 35^ The rationale for including mortality due to ACSC in this paper is an extension of the rationale for using unplanned ACSC hospitalisations as a marker of substandard primary care: if it is concerning when someone has an unplanned hospitalisation for a condition that should be sufficiently managed in primary care, it is even more concerning when someone dies of such condition.

## Methods

### Study design

The research was a population-based cohort data linkage study of people who took part in Scotland’s 2011 Census and were at least 18 years old on census day (27 March 2011). The study was focused on adults only because health conditions often have different prevalence in children and adults, and the primary care system in Scotland (as in many other countries) is arranged somewhat differently for children than adults. Therefore, including children in the study would have required a separate analysis, which was beyond the scope of the current project.

The two study cohorts consisted of all adults with ID (n=16 840) and a 15% randomly selected comparison sample from the general population (n=5 66 074). The sample size for the comparison sample was determined using the Scottish crude annual incidence rates and mortality rates for bowel cancer, oesophageal cancer and uterine cancer as examples, and previously published standardised incidence ratios and standardised mortality ratios for people with ID.^36^ The follow-up period ended on 31 December 2019. Census data was linked to individual-level data about hospitalisations and deaths.

The ID population included those with co-occurring autism, while the general population cohort excluded people with ID or autism. The individual’s ID status was determined from census data; one of the questions regarded disabilities and the list included ‘learning disability’ (the term much more widely used in the UK than ‘intellectual disability’, but with the same meaning). Census information was provided by a proxy where the respondent was not capable of completing the form. People who self-identified or proxy-identified as not having ID but whose death certificate mentions ID (International Classification of Diseases, 10th Revision codes F70, F71, F72, F73, F78, F79, F84) were excluded (n=30). People who died on the census day were also excluded. Unplanned ACSC hospitalisations were identified in hospital data using a codelist and specification provided by National Health Service (NHS Digital^37^ (online supplemental file 1).

A detailed description of Census 2011 methodology can be found in the National Records of Scotland (NRS) general report.^38^ As with other censuses, the 2011 Census aimed to include all members of the population rather than a sample. Data for 6% of the population who did not return the census form were imputed by the Census team. (As such, no weighting scheme is supposed to be applied in the analysis of census records). However, our study excluded those imputed records, as there was too much uncertainty that someone with imputed ID status really had ID. Where the census form was returned by the respondent with missing key demographic characteristics (age, sex, etc), NRS imputed missing values. However, we could not evaluate the extent of missingness/imputation, as the NRS did not publish relevant information. There were no missing cases on such variables in our dataset. The area deprivation variable was not subject to imputation by the NRS and had a large proportion of missing values (table 1).

**Table 1:** Age, sex and area deprivation profile of adults on census day, by ID status

	No ID	ID
Age		
Mean	49.0	43.9
Median	48	44
P25	34	29
P75	63	55
IQR	29	26
N	566 074	16 840
Sex (%)		
Males	47.2	55.7
Females	52.8	44.3
N	566 074	16 840
SIMD* decile (mean)	5.1	4.5
N	64 228	2874

* Scottish Index of Multiple Deprivation, a relative measure of deprivation across nearly 7000 small areas in Scotland (https://www.gov.scot/collections/scottish-index-of-multiple-deprivation-2020). Decile 1 represents the most deprived areas and decile 10 represents the least deprived areas.

ID, intellectual disabilities; N, Number of nonmissing records in the analysis dataset; P25, 25th percentile; P75, 75th percentile.

As the linked dataset did not contain primary care diagnoses, it was not possible to use it to calculate the prevalence of ACSC. An alternative dataset was therefore used to obtain prevalence rates (see below).

### Data

The three datasets used for this project (Scotland’s 2011 Census, hospital admissions, death certificate data) were provisioned by Electronic Data Research and Innovation Service (eDRIS, part of Public Health Scotland) via the Scottish National Safe Haven, a platform for researchers working with large databases of electronic health records (https://www.publichealthscotland.scot/services/data-research-and-innovation-services/electronic-data-research-and-innovation-service-edris/national-safe-haven-nsh/). The hospital admission dataset (SMR01) is described in detail in the National Data Catalogue (https://www.ndc.scot.nhs.uk/Data-Dictionary/SMR-Datasets/SMR01-General-Acute-Inpatient-and-Day-Case/). The death certificate data is maintained by the NRS. The datasets were merged by the authors using unique anonymised personal identifiers provided by eDRIS.

The prevalence of specific ACSC among the ID population in Scotland is unknown, and we did not have access to primary care data from which it could be estimated. We therefore used data from England, specifically the NHS data series ‘Health and Care of People with Learning Disabilities’ (https://digital.nhs.uk/data-and-information/publications/statistical/health-and-care-of-people-with-learning-disabilities), which is aggregate level. We regard the Scottish and English ID populations to be close enough in character for it to not be an issue for our analysis. While only 6 of 19 ACSC conditions are covered by this data source, collectively they are responsible for over half of all unplanned ACSC hospitalisations in Scotland (see below). We calculated prevalence point estimates and 95% CIs ourselves.

### Data analysis

#### Unplanned ACSC hospitalisations

The exposure was ID status, and the outcome was unplanned hospital admission that met the NHS-provided specification requirements for one of the ACSC.^37^ Note that while most ACSC diagnoses in this specification are based on a given ACSC being recorded as ‘primary’ diagnosis, 5 of the 19 ACSC (influenza and pneumonia, other vaccine preventable, chronic obstructive pulmonary disease (COPD), diabetes complications, gangrene) can meet the specification when they are recorded as non-primary diagnosis. For this reason, there is no ground for making a distinction between ‘hospitalisation due to ACSC’ and ‘hospitalisation with ACSC’ based on the diagnostic position.

The analysis focused on incidence rate ratios, both crude (IRR) and age-sex standardised (SIR). The IRR is the ratio of the incidence rate in an exposed group (here, people with ID) to the incidence rate in an unexposed (or comparison) group:

IRR=incidence rate in exposed group/incidence rate in unexposed group,

where an incidence rate is typically expressed as:

incidence rate=number of new events/total person-time at risk

The interpretation of IRR is as follows: IRR=1: no difference in incidence rates between groups; IRR>1: higher incidence rate in the exposed group; IRR<1: lower incidence rate in the exposed group. The formula for CI around IRR was as follows: when IRR is defined as (*a*/*N*1)/(*b*/*N*o), and *p*l and *pu* are the exact CI of the binomial probability for observing a successes in M1 trials, the exact CI for the incidence ratio is (pl*N*0)/((1 − *p*l)*N*1) to (p*uN*o)/((1 − *pu*)*N*1).^39^

SIR—often referred to as the standardised incidence rate ratio—is a measure used to compare the observed number of incident cases in a study population with the number expected if that population experienced the same incidence rates as a reference (standard) population. The SIR is defined as:

SIR=observed number of cases/ expected number of cases

The expected number of cases is calculated by applying age-specific, sex-specific or otherwise stratum-specific incidence rates from the standard population to the corresponding person-time in the study population, and then summing across strata. In our analysis, the standard (reference) population for age-sex adjustment was people without ID in the analysis dataset.

The interpretation of SIR is as follows: SIR=1: observed incidence equals that expected based on the standard population; SIR>1: higher incidence than expected; SIR<1: lower incidence than expected. The 95% CI around the SIR was calculated as follows: if O denotes ‘observed number of events’, the lower bound is SIR×exp(−1.96/sqrt(O)), and the upper bound is SIR×exp(+1.96/sqrt(O)).^39^

With regard to addressing the potential confounding, the two most important confounders—age and sex—have been adjusted for through age-sex standardisation of rates and ratios. Ethnicity could not be controlled for as we did not have relevant data. Adjustment for economic position (proxied by Scottish Index of Multiple Deprivation (SIMD)) was purposefully not done; poverty is both an outcome of ID as well as its determinant,^40 41^ and outcomes of the exposure should not be included in the causal path, as they may bias the effect of the exposure.^42 43^

Rates, rate ratios and their 95% CIs were calculated using the ‘stir’ command in Stata V.17. We also report cumulative incidences as these are easier to understand for audiences without background in epidemiology. Additionally, they may be more useful for evaluating the burden on the healthcare system, as for this, it does not matter whether two hospitalisations come from the same person or from two persons. (This matters in the calculation of time at risk for rates, though). However, as people with ID live on average shorter than people without ID,^44^ and obviously people who leave the cohort cannot be hospitalised, incidence rates are generally a more appropriate measure in this context than cumulative incidences.

#### Deaths due to ACSC and deaths involving ACSC

The analysis of mortality was somewhat different to the analysis of unplanned hospitalisations. While primarily interested in rate ratios, it was informative to investigate what proportion of all deaths in each population were due to ACSC and to distinguish between ‘deaths due to ACSC’, where ACSC is reported as main-cause (‘underlying cause’) of death on the death certificate and from ‘deaths involving ACSC’, where ACSC is reported in any position on the death certificate (‘all-cause’).

In all analyses, statistical significance was defined at the conventional 5% level. Differences in group proportions were tested via the ‘prtesti’ function in Stata V.17 (which uses a large-sample, normal-approximation z-test for proportions^39^) while differences in means were investigated via t-test (with normality evaluated through a visual inspection of the histogram), except for analyses that adjusted for ACSC prevalence; we examined the overlap of 95% CIs there instead. Statistical significance on ratios was determined by examining whether the 95% CI crossed 1. The analyses were conducted in Stata V.17. The code is available from the authors on request. Additional tables for specific age groups (18–54, 55+, 18–64, 65+) and for men and women are also available on request.

### Patient and public involvement

The Scottish Learning Disabilities Observatory has a steering committee which provides strategic direction and oversight of all of the observatory’s work, including this project. The steering committee includes people with learning disabilities from ‘People First’, a national group of self-advocates with learning disabilities.

Adults with learning disabilities will be involved in the production of ‘Easy Read’ materials based on the findings of this study.

## Results

### Characteristics of the study population

The size of the whole adult ID population on census day was 16 840. The size of the 15% general population random sample was 566 074 adults. The total number of person-years was 4 818 262, of which 134 769 in the ID cohort and 4 683 493 in the general population.

Adults with ID were, on average, younger than adults without ID (table 1). The ID population had relatively more males than females in comparison to the general population. On average, people with ID tended to live in somewhat more deprived areas than people without ID.

### Unplanned ACSC hospitalisations

#### Cumulative incidence

The cumulative incidence of unplanned hospitalisations for ACSC was overall similar for the ID and general population. Around 5% of all unplanned hospitalisations were due to ACSC, regardless of ID status (online supplemental table A1). Around 3% of unique persons had one or more unplanned ACSC hospitalisation, again regardless of ID status (table 2). Seizures and epilepsy, COPD and influenza and pneumonia were responsible for half of all unplanned ACSC hospitalisations among people without ID, and for 55% among people with ID.

**Table 2:** Statistics on adults alive on census day who had at least one unplanned hospitalisation due to ACSC during the study period, by ID status and ACSC, 2011–2019 (sorted by decreasing order of ‘Number of persons’ in ID)

ACSC	No ID		ID		P value on the group difference in ‘Percent of baseline population’*
	Number of persons	Per cent of baseline population (95% CI)	Number of persons	Per cent of baseline population (95% CI)	
Any ACSC	16375†	2.89 (2.85 to 2.94)	470	2.79 (2.55 to 3.05)	0.437
Influenza and pneumonia	3641	0.64 (0.62 to 0.66)	114	0.68 (0.56 to 0.81)	0.589
Seizures and epilepsy	2580	0.46 (0.44 to 0.47)	81	0.48 (0.38 to 0.60)	0.632
COPD	2399	0.42 (0.41 to 0.44)	61	0.36 (0.28 to 0.47)	0.225
Cellulitis	1870	0.33 (0.32 to 0.35)	49	0.29 (0.22 to 0.38)	0.379
Ear, nose and throat infections	1477	0.26 (0.25 to 0.27)	40	0.24 (0.17 to 0.32)	0.557
Congestive heart failure	1546	0.27 (0.26 to 0.29)	38	0.23 (0.16 to 0.31)	0.244
Angina	1001	0.18 (0.17 to 0.19)	38	0.23 (0.16 to 0.31)	0.139
Dehydration and gastroenteritis	1027	0.18 (0.17 to 0.19)	34	0.20 (0.14 to 0.28)	0.539
Diabetes complications	994	0.18 (0.16 to 0.19)	30	0.18 (0.12 to 0.25)	0.938
Asthma	1057	0.19 (0.18 to 0.20)	29	0.17 (0.12 to 0.25)	0.667
Pyelonephritis	481	0.08 (0.08 to 0.09)	18	0.11 (0.06 to 0.17)	0.338
Iron deficiency anaemia	429	0.08 (0.07 to 0.08)	11	0.07 (0.03 to 0.12)	0.626
Perforated/bleeding ulcer	182	0.03 (0.03 to 0.04)	7	0.04 (0.02 to 0.09)	0.504
Gangrene	309	0.05 (0.05 to 0.06)	6	0.04 (0.01 to 0.08)	0.297
Dental conditions	286	0.05 (0.04 to 0.06)	5	0.03 (0.01 to 0.07)	0.233
Hypertension	220	0.04 (0.03 to 0.04)	5	0.03 (0.01 to 0.07)	0.550
Pelvic inflammatory disease	108	0.02 (0.02 to 0.02)	<5		
Other vaccine preventable	39	0.01 (0.00 to 0.01)	<5		
Nutritional deficiencies	<5		<5		

* z-test for proportions using the ‘prtesti’ function in Stata V.17.

† Rounded to the nearest 5 to avoid disclosure on the number of nutritional deficiencies.

Those who had at least one unplanned ACSC hospitalisation had on average just over two ACSC hospitalisation spells (a ‘continuous inpatient spell’ is a continuous period of care within the NHS, regardless of any transfers which may take place) in the follow-up period, in both populations (online supplemental table A5). COPD was the ACSC with the highest mean number of spells per person. Overall means were similar in both cohorts, except for diabetes complications which had a noticeably lower mean in the ID cohort (1.4 vs 2.1).

The average length of hospital stay was similar between the two cohorts (online supplemental table A3).

### Incidence rates and ratios

The crude incidence rates of unplanned hospitalisations for ‘any ACSC’ were similar in both ID and general populations, with the difference not being statistically significant (online supplemental table A2). The age-sex standardised incidence rate for ID was similar to the crude rate.

As would be expected from cumulative incidence findings, seizures and epilepsy, COPD and influenza and pneumonia had the highest incidence rates in both cohorts, and incidence rates were similar between people with ID and the general population.

Most crude and age-sex SIRs were close to 1.0 (table 3), which is unsurprising in the light of the fact that most incidence rates were similar in both populations. Only one SIR was statistically significantly different from 1.0: in the case of diabetes complications, it was lower than 1.0.

**Table 3:** Crude IRR and SIR of unplanned adult hospitalisations due to an ACSC per 1000 person-years, ID population (numerator) compared with No ID population (denominator), 2011–2019 (sorted by decreasing SIR)

ACSC	IRR	IRR 95% CI	SIR	SIR 95% CI
Any ACSC	1.01	(0.94 to 1.07)	0.98	(0.91 to 1.05)
Perforated/bleeding ulcer	1.26u	(0.50 to 2.64)u	1.44	(0.66 to 3.14)
Pyelonephritis	1.22u	(0.73 to 1.93)u	1.18	(0.74 to 1.90)
Seizures and epilepsy	1.14	(0.98 to 1.31)	1.17	(1.00 to 1.36)
Influenza and pneumonia	1.11	(0.93 to 1.31)	1.09	(0.91 to 1.30)
Dehydration and gastroenteritis	1.14	(0.81 to 1.57)	1.07	(0.77 to 1.49)
COPD	1.05	(0.90 to 1.22)	1.03	(0.88 to 1.21)
Ear, nose and throat infections	1.06	(0.79 to 1.38)	1.01	(0.76 to 1.35)
Asthma	1.03	(0.79 to 1.33)	0.95	(0.73 to 1.23)
Angina	1.08	(0.77 to 1.48)	0.94	(0.68 to 1.29)
Cellulitis	0.83	(0.63 to 1.07)	0.86	(0.65 to 1.15)
Congestive heart failure	0.91	(0.69 to 1.19)	0.84	(0.64 to 1.11)
Hypertension	0.71u	(0.23 to 1.68)u	0.75	(0.31 to 1.83)
Iron deficiency anaemia	0.84u	(0.43 to 1.49)u	0.72	(0.40 to 1.30)
Gangrene	0.67u	(0.27 to 1.40)u	0.61	(0.28 to 1.31)
Diabetes complications	0.66	(0.48 to 0.90)	0.59	(0.43 to 0.81)
Dental conditions	0.57u	(0.18 to 1.34)u	0.53	(0.21 to 1.29)
Other vaccine preventable				
Nutritional deficiencies				
Pelvic inflammatory disease				

Note: letter ‘u’ indicates that the figure may not be reliable due to the small number of cases (between 5 and 20).

ACSC, ambulatory care sensitive conditions; COPD, chronic obstructive pulmonary disease; ID, intellectual disabilities; IRR, incidence rate ratios; SIR, standardised incidence ratio.

When the populations were broken down by age bands and sex, however, some important patterns started to appear (table 4). Among adults aged under 55, the SIR for ‘any ACSC’ was statistically significantly above 1.0. In contrast, the SIR for ‘any ACSC’ was statistically significantly below 1.0 among adults aged 55+. With regard to specific ACSC conditions, among adults aged under 65, the SIR for seizures and epilepsy was statistically significantly above 1.0, while the SIR for asthma was significantly above 1.0 among adults aged under 55. SIR for congestive heart failure was significantly below 1.0 among people aged 55+. SIR for influenza and pneumonia was significantly above 1.0 among adult women aged under 55, and SIR for COPD was significantly above 1.0 among adult men aged under 65.

**Table 4:** Statistically significant SIRs and 95% CIs on unplanned ACSC hospitalisations among adults aged 18+, by age group

ACSC	Age group			
	18+	Under 55*	Under 65	55+
Any		1.11 (1.03 to 1.20)		0.77 (0.66 to 0.89)
Diabetes complications†	0.59 (0.43 to 0.81)			
Convulsions and epilepsy			1.27 (1.08 to 1.48)	
Asthma		1.40 (1.07 to 1.85)		
Congestive heart failure				0.42 (0.21 to 0.86)
Influenza and pneumonia		Females: 1.32 (1.01 to 1.74)		
COPD			Males: 1.27 (1.03 to 1.55)	

* The SIRs in this column were not significant when age under 65 (or 18+) was used.

† Repeated from table 3 for completeness.

ACSC, ambulatory care sensitive conditions; COPD, chronic obstructive pulmonary disease; SIR, standardised incidence ratio.

As stated earlier, the aim of this study was to consider IRRs not by themselves but in the context of population prevalence. All results presented above table 5 present prevalence ratios for six ACSC.

**Table 5:** Crude and standardised prevalence ratios, ID adults (numerator) compared with no-ID adults (denominator), by ACSC, 2021–2022, England

ACSC	PR	PR 95% CI	Age-sex SPR	95% CI for age-sex SPR
COPD	0.66	(0.63 to 0.69)	0.96	(0.91 to 1.01)
Asthma	1.54	(1.52 to 1.57)	1.58	(1.55 to 1.61)
Convulsions and epilepsy	24.01	(23.71 to 24.31)	28.09	(27.74 to 28.45)
Congestive heart failure	0.98	(0.93 to 1.02)	1.4	(1.33 to 1.48)
Hypertension	0.74	(0.73 to 0.75)	1.01	(1.00 to 1.03)
Diabetes*	1.42	(1.39 to 1.44)	1.78	(1.75 to 1.81)
Non-type 1 diabetes	1.39	(1.37 to 1.42)	1.79	(1.76 to 1.82)
Type 1 diabetes	1.79	(1.69 to 1.90)	1.67	(1.57 to 1.77)

Health and Care of People with Learning Disabilities data series (https://digital.nhs.uk/data-and-information/publications/statistical/health-and-care-of-people-with-learning-disabilities).

* To allow for comparisons with diabetes complications in our hospitalisations and deaths data, figures for diabetes have been calculated by us by aggregating non-type 1 and type 1 diabetes. This is not ideal as non-type 1 and type 1 can co-occur. However, this happens relatively rarely.^50^

ACSC, ambulatory care sensitive conditions; COPD, chronic obstructive pulmonary disease; ID, intellectual disabilities; PR, prevalence ratio; SPR, standardised PR.

To adjust our ACSC hospitalisations analysis for the prevalence of ACSCs we compared 95% CIs of the hospitalisations (SIR) (last column in table 3) with 95% CIs of the prevalence (standardised prevalence ratio (SPR)) (last column in table 5). Where those CIs do not overlap, the difference between people with and without ID is statistically significant, and people with ID are more likely to have unplanned ACSC hospitalisations (if the lower bound of the SIR is larger than the upper bound of the SPR) or are less likely to have unplanned ACSC hospitalisations (if the upper bound of the SIR is smaller than the lower bound of the SPR).

The SIR for hospitalisations was statistically significantly lower than the SPR in the case of asthma (particularly among men), convulsions and epilepsy, congestive heart failure and diabetes complications among all adults. Additionally, in the 18–54 age range, SIR was lower than SPR for congestive heart failure and COPD.

### Deaths among adults

#### Cumulative mortality

The proportion of all adults alive on census day who died during the follow-up period was 11.4% in the No ID cohort and 16.7% in the ID cohort. With regards to ACSC ‘main-cause’, the proportions were 0.85% of the No ID and 1.3% of the ID (table 6). There were approximately 3.5 times more deaths involving ACSC (‘all-cause’): 3.2% of No ID adults and 4.5% of adults in the ID cohort (online supplemental table A6).

**Table 6:** Number and per cent of adults alive on census day who died during the study period and whose death certificate mentions ACSC as 'main-cause of death’, by ID status, 2011–2019 (sorted by decreasing order of ID n)

ACSC	No ID			ID			Stat. sig. (p value) on group difference in ‘Percent of baseline population’*
	n	Per cent of baseline population (95% CI)	Per cent of deaths due to ACSC	n	Per cent of baseline population (95% CI)	Per cent of deaths due to ACSC	
Any ACSC	4829	0.85 (0.83 to 0.88)	100.0	225	1.34 (1.17 to 1.52)	100.0	<0.001
Convulsions and epilepsy	92	0.02 (0.01 to 0.02)	1.9	93	0.55 (0.45 to 0.68)	41.3	<0.001
COPD	3758	0.66 (0.64 to 0.69)	77.8	77	0.46 (0.36 to 0.57)	34.2	0.001
Asthma	130	0.02 (0.02 to 0.03)	2.7	13	0.08 (0.04 to 0.13)	8.9	<0.001
Congestive heart failure	384	0.07 (0.06 to 0.07)	8.0	10	0.06 (0.03 to 0.11)	8.9	0.677
Influenza and pneumonia	130	0.02 (0.02 to 0.03)	2.7	10	0.06 (0.03 to 0.11)	8.9	0.003
Pyelonephritis	25	0.00 (0.00 to 0.01)	0.5	5	0.03 (0.01 to 0.07)	4.4	<0.001
Hypertension	159	0.03 (0.02 to 0.03)	3.3	8	0.05 (0.02 to 0.09)	3.6	0.142
Cellulitis	100	0.02 (0.01 to 0.02)	2.1	5	0.03 (0.01 to 0.07)	2.2	0.252
Dehydration and gastroenteritis	30	0.01 (0.00 to 0.01)	0.6	<5			
Angina	12	0.00 (0.00 to 0.00)	0.2	<5			
Gangrene	<5			<5			
Pelvic inflammatory disease	<5			<5			
Nutritional deficiencies	<5			<5			
Ear, nose and throat infections	<5			<5			
Dental conditions	<5			<5			
Diabetes complications	<5			<5			
Iron deficiency anaemia	<5			<5			
Perforated/bleeding ulcer	<5			<5			
Other vaccine preventable	<5			<5			

* z-test for proportions using the ‘prtesti’ function in Stata V.17.

ACSC, ambulatory care sensitive conditions; COPD, chronic obstructive pulmonary disease; ID, intellectual disabilities.

Of those adults who died during the follow-up period, 7.5% of people without ID died due to ACSC (main-cause) cf. 7.8% of people with ID. The proportion of all deaths that involved ACSC (all-cause) was 28.4% in the No ID cohort and 26.5% among people with ID.

The mean age of adults at death was 77 in the No ID cohort and 63 in the ID cohort. The mean age of adults at death due to ACSC was 78 in No ID and 62 in the ID cohort.

The vast majority (75%) of main-cause ACSC deaths in ID were caused by just two ACSCs: convulsions and epilepsy (41%) and COPD (34%) (table 6). The picture was different among adults without ID, with COPD responsible for 78% of deaths and very few convulsions and epilepsy deaths (2%). Among women with ID, there were somewhat more ACSC deaths due to asthma, influenza and pneumonia and congestive heart failure than among men with ID (20% vs 8% of all ACSC deaths). Among older (55+) people with ID, COPD overtakes convulsions and epilepsy as the leading cause of ACSC deaths (45% vs 25%). Among older women (55+), the proportion of main-cause deaths due to asthma gradually increases to the point of becoming second-leading in the 65+ age group. With regard to all-cause ACSC deaths, apart from convulsions and epilepsy and COPD, congestive heart failure and hypertension are major all-cause causes of ACSC death, in both ID and non-ID populations.

### Mortality rates and ratios

People with ID had considerably higher ACSC mortality rates (both crude and age/sex-standardised) than people without ID (online supplemental table A4).

Without taking ACSC prevalence into account, main-cause age-sex standardised mortality ratios (SMR) were statistically significantly higher on asthma (particularly among women), cellulitis, convulsions and epilepsy, COPD, hypertension, influenza and pneumonia (particularly among women) and pyelonephritis (particularly among women) (table 7). Additionally, in the 18–64 age group, the SMR on congestive heart failure was higher and statistically significant (particularly among women).

**Table 7:** Adult ACSC mortality ratios, cases with ACSC mentioned as ‘main cause of death’ on the death certificate, ID cohort (numerator) compared with No ID cohort (denominator), 2011–2019 (sorted by decreasing SMR)

ACSC	MR	MR 95% CI	SMR	SMR 95% CI
Any ACSC	1.62	(1.41 to 1.85)	2.54	(2.19 to 2.95)
Convulsions and epilepsy	35.13	(26.05 to 47.38)	39.99	(29.61 to 53.99)
Pyelonephritis	6.95u	(2.08 to 18.48)	12.10u	(4.53 to 32.30)
Asthma	3.48u	(1.80 to 6.15)	6.30u	(3.38 to 11.73)
Influenza and pneumonia	2.67u	(1.25 to 5.08)	4.31u	(2.11 to 8.82)
Hypertension	1.75u	(0.74 to 3.53)	4.08u	(1.94 to 8.58)
Cellulitis	1.74u	(0.55 to 4.19)	3.81u	(1.47 to 9.86)
Congestive heart failure	0.91u	(0.43 to 1.68)	1.64u	(0.82 to 3.27)
COPD	0.71	(0.56 to 0.89)	1.32	(1.04 to 1.69)
Dehydration and gastroenteritis				
Gangrene				
Other vaccine preventable				
Pelvic inflammatory disease				
Angina				
Iron deficiency anaemia				
Perforated/bleeding ulcer				
Diabetes complications				
Dental conditions				
Ear, nose and throat infections				
Nutritional deficiencies				

Note: letter ‘u’ indicates that the figure may not be reliable due to the small number of cases (between 5 and 20).

ACSC, ambulatory care sensitive conditions; COPD, chronic obstructive pulmonary disease; ID, intellectual disabilities; MR, mortality ratio; SMR, standardised MR.

When ACSC prevalence was taken into account, the above findings for asthma, convulsions and epilepsy, COPD, hypertension and congestive heart failure remained unchanged. No prevalence data was available for cellulitis, influenza and pneumonia, and pyelonephritis.

Moving on to all-cause mortality, nominally SMRs were higher and statistically significant on asthma, cellulitis, convulsions and epilepsy, congestive heart failure, COPD, dehydration and gastroenteritis, gangrene, hypertension, influenza and pneumonia, and pyelonephritis (online supplemental table A7). Taking prevalence into account, the findings were unchanged with regards to asthma, congestive heart failure, COPD and hypertension. SMR for convulsions and epilepsy was no longer statistically significant. No prevalence data was available for cellulitis, dehydration and gastroenteritis, gangrene, influenza and pneumonia, and pyelonephritis.

## Discussion

### Unplanned hospitalisations

It is clear that unplanned ACSC hospitalisations in Scotland constitute a non-negligible proportion of all hospitalisations (5%), regardless of ID status, burdening the healthcare budget in times of ever-increasing demand from an ageing population. As for what our findings tell us about the quality of primary care or the access to it, on their own, the higher SIR for ‘any ACSC’ among people aged under 55 and higher SIRs (among certain age groups and/or genders) for convulsions and epilepsy, asthma, influenza and pneumonia, and COPD would suggest a negative story about how primary care in Scotland works for people with ID (particularly those aged under 55), with respect to those specific conditions. In contrast, the lower SIR on diabetes complications would suggest a positive story with regards to that specific condition.

However, as argued in the introduction, it is more appropriate to consider IRR not by themselves but in the context of population prevalence. When this is considered, not only do the negative findings about seizures and epilepsy, asthma, influenza and pneumonia, and COPD disappear, but the picture turns out to be positive: the hospitalisation ratio (SIR) is lower than the prevalence ratio (SPR); the difference is statistically significant as CIs do not overlap. Furthermore, the picture turns out to be positive in the 18–54 age range with regards to congestive heart failure. Therefore, in the case of these conditions as well as diabetes complications, the primary care system in Scotland seems to work at least as well for people with ID as for people without ID.

Having said that, it is possible that the picture that we obtained from our data is biased towards positive. We are aware of there being anecdotal knowledge among some family members of people with ID suggesting that the effective hospital admission threshold may be higher for people with ID than those without (eg, due to diagnostic overshadowing). Should this anecdotal knowledge be correct, the number of unplanned hospital admissions for ACSC among people with ID would have been higher than actually recorded in the data, and the risk in that group would have been higher than reported here.

The higher mortality from ACSC experienced by people with ID also points toward the challenges involved in recognising and managing ACSC experienced by people with ID. Previous studies have highlighted that physical health conditions often go unrecognised for some time, leading to the delayed presentation to services and diagnosis, which has been linked to premature death of people with ID.^45^ These difficulties in recognising physical health conditions in adults with ID have led some countries to introduce annual health checks for adults with ID. However, it is important to strengthen the capacity of carers and support staff to recognise undiagnosed physical health conditions and train mainstream healthcare professionals to assess and manage physical health conditions of adults with ID.^46^

Our findings diverge from those of studies in England^19 27 28^ in that they found a much higher risk of unplanned ACSC hospitalisations among people with ID. In particular, Glover *et al*^19^ found the standardised risk to be five times higher, while we found the risk to be only slightly higher (among people aged under 55, the standardised ratio was 1.11), or similar. A question arises as to why this is the case. The make-up of the healthcare system is very similar in England and Scotland, and therefore it should not be the factor behind different results. In our view, it is likely to be due to methodological differences between our study and those other ones. The key difference is that we used self-reporting or proxy-reporting to identify the presence of ID. The most recent English study by Glover *et al*^19^ used the learning disability register for this purpose, while the two earlier English studies by Hosking *et al* and by Glover and Evison^27 28^ relied on diagnostic codes to identify ID. It is likely that some people with ID were captured by the self/proxy report method but would be missed by the latter methods. In the opposite direction, perhaps some people with ID were missed by the census method while they would be captured by the latter methods. (Interestingly, the census and the learning disability register methods both identified around 0.5% of the respective populations as having ID). Unfortunately, as Scotland does not have a learning disability register (and does not give researchers access to patient-level primary care data) and England does not identify ID in the national census, not even an ‘informed guess’ is possible as to how big or small the overlap might be.

One explanation of the difference in results could therefore be that the overlap between the two methods is poor, and our study captured quite a different subpopulation of people with ID than the cited English studies. Perhaps if all people with ID were correctly identified—closer to 2% of the population^47^ than 0.5%—the risk of people with ID having an unplanned ACSC hospitalisation would turn out to be higher than in our study but lower than in the English studies. Until more research is done, however, this is only a speculation.

It is also possible that methodological limitations of those other studies are responsible for some or most of the difference in findings. For example, the Glover & Evison study^28^ may have misclassified some people with milder ID as not having ID at all; the authors acknowledged that ‘we cannot be sure that the individuals we have identified are representative of people with learning disabilities as a whole, though we think they are probably reasonably representative of people with severe or profound learning disabilities’. Glover *et al*^19^ similarly acknowledge that they ‘probably missed many patients who have mild to moderate ID without obvious syndromic causes whose GPs have not recorded this’. The Hosking *et al* study^27^ may have also misclassified some patients; they included in the ID cohort not only those with diagnostic codes used by the Quality and Outcomes Framework (QOF) for learning disability but also those who had diagnoses of conditions ‘usually associated with ID’. (No detail is given as to what proportion of the ID cohort were identified through which avenue, and no sensitivity analysis is provided).

### Mortality

While the proportion of ACSC deaths in all deaths was similar in the two populations, our investigation of rates and ratios showed possible health inequalities for the population with ID. SMRs were higher than prevalence ratios for asthma, seizures and epilepsy, COPD, hypertension and congestive heart failure. In line with existing evidence on shorter life expectancy among people with ID,^44^ the cumulative mortality in our study was much higher among people with ID. We did not have prevalence data about other ACSCs, so it is possible that other conditions have SMRs higher than prevalence ratios.

Since the vast majority of ACSC deaths in ID were caused by just two ACSCs: seizures and epilepsy and COPD, this implies that the biggest gains in reducing mortality caused by ACSC could be achieved by targeting these two conditions. The Office for National Statistics regards epilepsy as ‘treatable’ and COPD as ‘preventable’.^48^

Also, with regards to seizures and epilepsy, it is worth highlighting that it is one of the four leading causes of unplanned ACSC hospitalisations in the general population, but very few deaths are due to it (table 6). Among people with ID, seizures and epilepsy produce both a lot of unplanned hospitalisations and a lot of deaths. This might suggest that when general practitioners treat an ID patient with seizures and epilepsy, they should be aware of this much higher risk of death.

### Implications for policy and practice

The study’s findings about unplanned ACSC hospitalisations seem to provide—at least at face value—a welcome exception from typically negative stories about the situation of people with ID. There are, however, three important qualifications that should be made. First, the findings do not mean that the primary care system in Scotland is objectively ‘good’ for people with ID; they suggest it is overall ‘not worse’ for people with ID than for people without ID. The authors regularly meet people with ID and their carers (in research and use-of-services contexts), and their testimonies indicate that there is still significant room for improvement.

A second, related qualification regards a point made in the introduction, that using ACSC evidence from different countries presents an opportunity for policy or practice ‘transfer’. The ratio of unplanned ACSC hospitalisation rates to ACSC prevalence could be used to identify which countries can learn from which. Countries with a worse (higher) ratio than Scotland’s could benefit from transferring some Scottish policy and practice solutions. On the other hand, Scotland could benefit from looking into primary care systems in countries with a better (lower) ratio.

Third, our findings about inequalities in ACSC mortality between people with ID and people without ID are not positive. More research is needed to understand why the picture of ACSC mortality is worse than the picture of unplanned ACSC hospitalisations.

### Future research

The findings of this study highlight the importance of future research to explore ways to reduce the need for hospitalisation for ACSC experienced by adults with ID and improve the outcomes when individuals do need to be hospitalised. Annual health checks for all adults with ID were introduced across Scotland and offer a naturalised experiment to examine whether health checks reduce hospitalisations for ACSC.^31^ Researchers should explore whether the increased mortality from ACSC after hospitalisation can be explained by delayed recognition of ACSC, leading to hospitalisation with more severe disease pathology.^49^ Finally, given the significant change in society and social and healthcare services since the COVID-19 pandemic, follow-up studies should consider using severe ACSC outcomes such as hospitalisation and mortality to examine whether there is evidence for ongoing improvements in the health of adults with ID.

### Strengths and limitations

The study’s main strength lies in the use of rigorous methodologies. The Census had a high response rate (94%) and the linkage of the Census to other datasets was carried out by a dedicated service (eDRIS) specialising in this kind of work, thus increasing our trust in the quality of the linkage. The dataset of hospital admissions and the dataset of death certificates are both long-standing and undergo high quality control. The list of ACSCs and the methodology for identification of ACSCs have been produced by the national healthcare provider, again increasing our confidence in the validity and reliability of the findings.

Another major strength of the study is that it took into account ACSC prevalence, unlike most of the previous studies.

The study’s biggest limitation was the small size of the ID population in Scotland. This meant that our CIs were often too wide to draw any conclusions. When CIs are wide, it is not possible to know whether differences in rates and ratios reflect the reality or are just due to random chance.

A further limitation was that we had prevalence data only for 6 out of 19 ACSCs. However, within those 6, 2 (convulsions and epilepsy, COPD) are the leading causes of unplanned ACSC hospitalisations in ID and 4 are the leading causes of all-cause mortality. Therefore, this limitation is relatively minor.

It is a limitation of the study that a sensitivity analysis could not be conducted. Logically, a sensitivity analysis would take the form of sourcing the person’s ID status from primary care records rather than from the Census, and linking it to hospital admissions and death data. However, as Scotland does not have an ID register and researchers are not given access to patient-level primary care records, such sensitivity analysis was not possible. This also entailed that the prevalence of ACSCs in the ID population in Scotland was not available and had to be sourced from England.

Where the census form was filled by an individual with ID rather than by proxy, it is possible that some responses to the question about ID were ‘false negatives’, perhaps due to misunderstanding of the question or due to respondents not wanting to admit that they had ID. To this extent, we may have misclassified some people with ID as members of the general population.

Another limitation of the study comes from the fact that the NRS have not published information that would allow us to evaluate the extent of item missingness (and imputation) on key demographic variables in the census and whether those variables could be considered missing completely at random. However, historically much effort has gone into strengthening census imputation procedures, which increases our confidence in the quality of imputation done to the dataset that we used.

It might have been beneficial to have a fuller socio-demographic profile of the ID and no-ID populations at the time of the census. Unfortunately, only the sex, age and SIMD variables were included in the dataset provided to us.

Lastly, we did not have access to information about the person’s ethnic identity. Hence, we could not investigate whether people with ID identifying as non-white had different rates of unplanned ACSC hospitalisations than white people with ID.

## Conclusions

The study has enhanced our knowledge and understanding of ACSC among people with ID in Scotland. It has established that after adjusting for disease prevalence, people with ID are no longer at a higher risk of having an unplanned ACSC hospitalisation. Our conclusion is that as far as unplanned ACSC hospitalisations are concerned, the primary care system in Scotland seems to work at least as well for people with ID as it does for the general population. It needs to be emphasised, however, that our findings on deaths due to ACSC are much less positive and suggest that this system does not work for people with ID as well as for people without it. Furthermore, the validity of our findings is reliant on the adequacy of identifying ID through self-reporting or proxy-reporting. Last but not least, our findings do not mean that there is room for complacency: 5% of all hospitalisations and 8% of deaths are due to ACSC and as such are potentially preventable. Large benefits in liveability and savings to the healthcare budget can be achieved by improvements in primary care.

## Supplementary material

10.1136/bmjopen-2025-105055 online supplemental file 1

10.1136/bmjopen-2025-105055 online supplemental file 2

## Data Availability

Data may be obtained from a third party and are not publicly available.
