# Supplementary material for: Hospitalisations and deaths due to ambulatory care sensitive conditions among adults with and without intellectual disabilities in Scotland: a cohort study
Source: BMJ Open. 2026 Mar 2;16(3):e105055. doi: 10.1136/bmjopen-2025-105055 (PMC12958873; doi:10.1136/bmjopen-2025-105055)
Supplement: online supplemental file 1 [file bmjopen-16-3-s001.pdf]

Appendix A

Data Sources

- All hospital data are sourced from HES Admitted Patient Care (APC) Commissioning Data Set. It uses published data from April 2013 onwards.
- Organisation Reference Data is sourced from Organisation Data Service (ODS) section in NHS Digital website.

ACSC episodes

Finished Consultant Episodes with Admission Method ‘Emergency’ (EPISTAT = 3 and ADMIMETH in '21', '22', '23', '24', '28','2A', '2B', '2C', '2D') where any of the following ICD10 codes are present:

ACSC name	ICD10 code	Description	Comments
Influenza and pneumonia	J10	Influenza due to identified influenza virus	In any diagnosis field; exclude people under 3 months or with any secondary diagnosis of D57
	J11	Influenza, virus not identified	
	J13	Pneumonia due to Streptococcus pneumoniae	
	J14	Pneumonia due to Haemophilus influenzae	
	J153	Pneumonia due to streptococcus, group B	
	J154	Pneumonia due to other streptococci	
	J157	Pneumonia due to Mycoplasma pneumoniae	
	J159	Bacterial pneumonia, unspecified	
	J168	Pneumonia due to other specified infectious organisms	
	J181	Lobar pneumonia, unspecified	
	J188	Other pneumonia, organism unspecified	
Other vaccine preventable	A35	Other tetanus	In any diagnosis field
	A36	Diphtheria	
	A37	Whooping cough	
	A80	Acute poliomyelitis	
	B05	Measles	
	B06	Rubella [German measles]	
	B161	Acute hep B with delta-agent (coinfectn) without hep coma	
	B169	Acute hep B without delta-agent and without hepat coma	
	B180	Chronic viral hepatitis B with delta-agent	
	B181	Chronic viral hepatitis B without delta-agent	
	B26	Mumps	
	G000	Haemophilus meningitis	
	M014	Rubella arthritis	
Asthma	J45	Asthma	Principal diagnosis only
	J46	Status asthmaticus	

Congestive heart failure	I110	Hypertensive heart disease with (congestive) heart failure	Principal diagnosis only; Exclude main operative procedures with OPCS4 codes of K0,K1,K2,K3,K4,K50,K52,K55,K56,K57, K60,K61,K66,K67,K68,K69,K71
	I50	Heart failure	
	J81	Pulmonary oedema	
Diabetes complications	E100-E108	Insulin-dependent diabetes mellitus	In any diagnosis field
	E110-E118	Non-insulin-dependent diabetes mellitus	
	E120-E128	Malnutrition-related diabetes mellitus	
	E130-E138	Other specified diabetes mellitus	
	E140-E148	Unspecified diabetes mellitus	
Chronic obstructive pulmonary disease	J20	Acute bronchitis	Principal diagnosis only; ICD-10: J20 only if there is a secondary diagnosis of J41, J42, J43, J44, J47
	J41	Simple and mucopurulent chronic bronchitis	
	J42	Unspecified chronic bronchitis	
	J43	Emphysema	
	J44	Other chronic obstructive pulmonary disease	
	J47	Bronchiectasis	
Angina	I20	Angina pectoris	Principal diagnosis only; Exclude cases with main operative procedure OPCS4 codes of A, B,C, D, E, F, G, H, I, J, K, L, M, N, O, P, Q, R, S, T, V, W, X0, X1, X2, X4, X5
	I240	Coronary thrombosis not resulting in myocardial infarction	
	I248	Other forms of acute ischaemic heart disease	
	I249	Acute ischaemic heart disease, unspecified	
Iron deficiency anaemia	D501	Sideropenic dysphagia	Principal diagnosis only
	D508	Other iron deficiency anaemias	
	D509	Iron deficiency anaemia, unspecified	
Hypertension	I10	Essential (primary) hypertension	Principal diagnosis only; Exclude cases with main operative procedure OPCS4 code of K0, K1, K2, K3, K4, K50, K52, K55, K56, K57, K60, K61, K66, K67, K68, K69, K71
	I119	Hypertensive heart disease without (congestive) heart failure	
Nutritional deficiencies	E40	Kwashiorkor	Principal diagnosis only
	E41	Nutritional marasmus	
	E42	Marasmic kwashiorkor	
	E43	Unspecified severe protein-energy malnutrition	
	E550	Rickets, active	
	E643	Sequelae of rickets	
Dehydration and gastroenteritis	E86	Volume depletion	Principal diagnosis only
	K522	Allergic and dietetic gastroenteritis and colitis	
	K528	Other specified noninfective gastroenteritis and colitis	
	K529	Noninfective gastroenteritis and colitis, unspecified	
Pyelonephritis	N10	Acute tubulo-interstitial nephritis	Principal diagnosis only
	N11	Chronic tubulo-interstitial nephritis	
	N12	Tubulo-interstitial nephritis not spec as acute or chronic	
	N136	Pyonephrosis	
Perforated/bleeding ulcer	K250-K252,	Gastric ulcer	Principal diagnosis only
	K254-K256		
	K260-K262	Duodenal ulcer	
	K264-K266		

	K270-K272	Peptic ulcer, site unspecified	
	K274-K276		
	K280-K282	Gastrojejunal ulcer	
	K284-K286		
Cellulitis	L03	Cellulitis	Principal diagnosis only. Exclude cases with main operative procedure OPCS codes of A, B, C, D, E, F, G, H, I, J, K, L, M, N, O, P, Q, R, S1, S2, S3, S41, S42, S43, S44, S45, S48, S49, T, V, W, X0, X1, X2, X4, X5. Exclude cases with operative procedure OPCS4 code S47 if there are any other operative procedure codes quoted.
	L04	Acute lymphadenitis	
	L080	Pyoderma	
	L088	Other spec local infections of skin and subcutaneous tissue	
	L089	Local infection of skin and subcutaneous tissue, unspecified	
	L88	Pyoderma gangrenosum	
	L980	Pyogenic granuloma	
Pelvic inflammatory disease	N70	Salpingitis and oophoritis	Principal diagnosis only
	N73	Other female pelvic inflammatory diseases	
	N74	Female pelvic inflammatory disorders in diseases EC	
Ear, nose and throat infections	H66	Suppurative and unspecified otitis media	Principal diagnosis only
	H67	Otitis media in diseases classified elsewhere	
	J02	Acute pharyngitis	
	J03	Acute tonsillitis	
	J06	Acute upper respiratory infections multiple and unsp sites	
	J312	Chronic pharyngitis	
Dental conditions	A690	Necrotizing ulcerative stomatitis	Principal diagnosis only
	K02	Dental caries	
	K03	Other diseases of hard tissues of teeth	
	K04	Diseases of pulp and periapical tissues	
	K05	Gingivitis and periodontal diseases	
	K06	Other disorders of gingiva and edentulous alveolar ridge	
	K08	Other disorders of teeth and supporting structures	
	K098	Other cysts of oral region, not elsewhere classified	
	K099	Cyst of oral region, unspecified	
	K12	Stomatitis and related lesions	
	K13	Other diseases of lip and oral mucosa	
Convulsions and epilepsy	G40	Epilepsy	Principal diagnosis only
	G41	Status epilepticus	
	R56	Convulsions, not elsewhere classified	
	O15	Eclampsia	
Gangrene	R02	Gangrene, not elsewhere classified	In any diagnosis field

Classifications

There are slight differences in the way the ACSC are classified. We use the following which is based on a report published by The Health Foundation and Nuffield Trust[1].

Category	Condition
Acute	Cellulitis
	Dehydration and gastroenteritis
	Dental conditions
	Ear, nose and throat infections
	Gangrene
	Nutritional deficiencies
	Pelvic inflammatory disease
	Perforated/bleeding ulcer
	Pyelonephritis
Chronic	Angina
	Asthma
	Chronic obstructive pulmonary disease
	Congestive heart failure
	Convulsions and epilepsy
	Diabetes complications
	Hypertension
	Iron deficiency anaemia
Vaccine-preventable	Influenza and pneumonia
	Other vaccine preventable

Spells

The unit of analysis for ACSC in this report is a continuous inpatient (CIP) spell. A CIP spell is a continuous period of care in hospital, regardless of any transfers which may take place; it may include more than one episode or provider. An episode is a continuous period of admitted patient care under one consultant within one healthcare provider. The length of stay (LoS) is the duration of a spell, i.e. days between admission and discharge. ACSC spells are those CIP spells where the first episode is an ACSC episode.

The method of discharge (DISMETH) is 1, 2 or 3 (Discharged on clinical advice, Self-discharged, or discharged by a mental health review tribunal, i.e. not died, a baby or still in hospital).

For the first episode of the CIP spell (EPIORDER=1):

- The patient classification is an ordinary admission only (CLASSPAT=1).
- The primary diagnosis doesn't relate to Obstetrics (DIAG_01 doesn't begin with 'O')
- It is a General episode (EPITYPE = 1)

[1] Ian Blunt. (2013). Focus on preventable admissions, Tends in emergency admissions for ambulatory care sensitive conditions, 2001 to 2013. *Quality Watch.* Retrieved from https://www.health.org.uk/publications/qualitywatch-focus-on-preventable-admissions on March 2019.

- The age at start of episode is between 0 and 120 (STARTAGE)
- Patient date of birth not 01/01/1900 or 01/01/1901 representing unknown (MYDOB)
- The sex of patient is male or female (SEX is 1 or 2)
- The Main specialty is not

For the last episode of the CIP spell:

- It is a general episode only (EPITYPE=1)
- The Main specialty is not Obstetrics, Midwifery or General Practice with Maternity Function (MAINSPEF) is not 501, 560 or 610

And CIP spells that belong to patients who have had cancer episodes or chemotherapy for cancer both within the reporting year or anywhere in the 365 days prior to admission are not considered ACSC spells (DIAG_01 – DIAG_20 not C00-C97, D37-D48 or Z51.1.

One ACSC condition is assigned to each spell. Conditions numbered 1 to15 in the list below use primary diagnosis at time of admission, so these conditions were assigned to the ACSC spells first. The last four conditions in the list can appear in any diagnosis position in the admission episode and therefore were assigned after the first 15, as only one ACSC condition has been assigned to a spell.

1. Asthma
2. Congestive heart failure
3. Chronic obstructive pulmonary disease
4. Angina
5. Iron deficiency anaemia
6. Hypertension
7. Nutritional deficiencies
8. Dehydration and gastroenteritis
9. Pyelonephritis
10. Perforated/bleeding ulcer
11. Cellulitis
12. Pelvic inflammatory disease
13. Ear, nose and throat infections
14. Dental conditions
15. Convulsions and epilepsy
16. *Gangrene*
17. *Diabetes complications*
18. *Influenza and pneumonia*
19. *Other vaccine preventable*

Readmissions

For purposes of this report, if a patient is admitted to hospital within 29 days from the previous discharge, this is considered a readmission. The previous admission must be an ACSC spell for the subsequent admission to be considered as an ACSC readmission. The number of days between discharge date (DISDATE) of the ACSC spell and the admission date (ADMIDATE) of the subsequent admission, irrespective of whether it is an ACSC spell or not, must be between 0 and 29 days for it to be considered a readmission.

NHS Digital

Ethnicity

The ethnicity of the patient, as specified by the patient is recorded using 18 possible values as defined in the 2001 census. In this report they have been grouped as shown below.

Ethnicity	Ethnicity group
A = British (White)	White
B = Irish (White)	White
C = Any other White background	White
D = White and Black Caribbean (Mixed)	Mixed
E = White and Black African (Mixed)	Mixed
F = White and Asian (Mixed)	Mixed
G = Any other Mixed background	Mixed
H = Indian (Asian or Asian British)	Asian or Asian British
J = Pakistani (Asian or Asian British)	Asian or Asian British
K = Bangladeshi (Asian or Asian British)	Asian or Asian British
L = Any other Asian background	Asian or Asian British
M = Caribbean (Black or Black British)	Black or Black British
N = African (Black or Black British)	Black or Black British
P = Any other Black background	Black or Black British
R = Chinese (other ethnic group)	Other
S = Any other ethnic group	Other
Z = Not stated	Not stated
99 = Not known (2013 onwards)	Not known

Organisation reference data

The Clinical Commissioning Group (CCG) used in this analysis is derived in HES. It contains the code for CCG of Responsibility. In HES, this is derived from postcode of patient's GP practice, but if that data is not available it uses postcode of patient's residence and if that data is not available it uses postcode of the provider to lookup the CCG code from the Postcode Directory which is available in ODS section in the NHS Digital website.

The Sustainability and Transformation Partnership (STP) codes are derived using reference data available in ODS section in NHS Digital website from CCG of responsibility present in HES.

Suppression

Data suppression is based on HES Disclosure Control Methodology for Hospital Episode Statistics for the Admitted Patient Care (APC) data set. For the counts of spells: Zeroes remain unchanged, counts between 1 and 7 (inclusive) are shown as 5 and all other counts are rounded to the nearest 5.
