# Supplementary material for: Hospitalisations and deaths due to ambulatory care sensitive conditions among adults with and without intellectual disabilities in Scotland: a cohort study
Source: BMJ Open. 2026 Mar 2;16(3):e105055. doi: 10.1136/bmjopen-2025-105055 (PMC12958873; doi:10.1136/bmjopen-2025-105055)
Supplement: online supplemental file 2 [file bmjopen-16-3-s002.pdf]

Appendix

Table A1. All adult hospitalisation spells* broken down by whether they were unplanned hospitalisations due to ACSC, by ID status, 2011-19 (%)

	ACSC hospitalisation		Total	N
	No	Yes		
No ID	94.8	5.2	100	579,917
ID	94.8	5.2	100	17,276

* A continuous inpatient (CIP) spell is a continuous period of care within the NHS, regardless of any transfers which may take place.

Table A2. Incidence rates of adult hospitalisations due to an ACSC per 1,000 person-years, by ID status and ACSC, 2011-19 (sorted by decreasing SIR in ID group)

ACSC	Crude incidence rates (IR)				ID incidence rates age-sex standardized to 'No ID' population	
	No ID		ID			
	IR	IR 95% CI	IR	IR 95% CI	SIR	SIR 95% CI
Any ACSC	6.07	(6.00, 6.14)	6.10	(5.71, 6.51)	5.94	(5.56, 6.35)
Convulsions and Epilepsy	1.17	(1.14, 1.20)	1.33	(1.15, 1.53)	1.36	(1.18, 1.57)
COPD	1.17	(1.14, 1.20)	1.23	(1.06, 1.43)	1.21	(1.04, 1.40)
Influenza and Pneumonia	0.90	(0.87, 0.92)	0.99	(0.84, 1.17)	0.98	(0.83, 1.15)
Cellulitis	0.50	(0.48, 0.52)	0.41	(0.32, 0.53)	0.43	(0.33, 0.55)
Asthma	0.41	(0.39, 0.43)	0.42	(0.33, 0.55)	0.39	(0.30, 0.51)
Ear, nose, and throat inflections	0.37	(0.35, 0.39)	0.39	(0.30, 0.51)	0.37	(0.29, 0.49)

Congestive Heart Failure	0.43	(0.41, 0.45)	0.39	(0.30, 0.51)	0.36	(0.27, 0.47)
Diabetes complications	0.44	(0.42, 0.46)	0.29	(0.22, 0.40)	0.26	(0.19, 0.36)
Dehydration and Gastroenteritis	0.24	(0.22, 0.25)	0.27	(0.20, 0.37)	0.25	(0.18, 0.35)
Angina	0.26	(0.24, 0.27)	0.28	(0.20, 0.38)	0.24	(0.17, 0.34)
Pyelonephritis	0.11	(0.10, 0.12)	0.13u	(0.08, 0.21)u	0.13u	(0.08, 0.20)u
Iron deficiency anaemia	0.10	(0.09, 0.11)	0.08u	(0.05, 0.15)u	0.07u	(0.04, 0.13)u
Perforated/bleeding ulcer	0.04	(0.03, 0.04)	0.05u	(0.02, 0.10)u	0.06u	(0.03, 0.11)u
Gangrene	0.07	(0.07, 0.08)	0.05u	(0.02, 0.10)u	0.04u	(0.02, 0.10)u
Hypertension	0.05	(0.04, 0.06)	0.03u	(0.01, 0.08)u	0.04u	(0.02, 0.09)u
Dental conditions	0.06	(0.05, 0.07)	0.03u	(0.01, 0.08)u	0.03u	(0.01, 0.08)u
Pelvic Inflammatory Disease	0.03	(0.02, 0.03)				
Other Vaccine Preventable	0.01	(0.01, 0.02)				
Nutritional deficiencies						

Note: letter ‘u’ indicates that the figure may not be reliable due to the small number of cases (between 5-20).

Table A3. Average length of unplanned hospital stays due to ACSC, by ID status and ACSC, 2011-19 (measured in days; stays of adults on Census 2011 day; length refers to hospital spells. Sorted by decreasing mean length of stay in the ID group)

ACSC	No ID			ID		
	Number of spells	median	mean	Number of spells	median	mean
Any ACSC	30690*	1.0	2.5	924	1.0	2.6
Dental conditions	298	1.0	1.7	5	1.0	13.0

Gangrene	351	3.0	7.8	7	4.0	4.7
Influenza and Pneumonia	4342	1.0	3.2	143	1.0	3.8
Cellulitis	2395	1.0	3.1	59	1.0	3.7
Perforated/bleeding ulcer	187	1.0	2.4	7	2.0	3.4
Diabetes complications	2133	1.0	3.4	42	1.0	3.3
COPD	5663	1.0	2.5	177	1.0	2.9
Congestive Heart Failure	2061	1.0	3.3	56	1.0	2.4
Dehydration and Gastroenteritis	1147	1.0	3.2	39	1.0	2.1
Pyelonephritis	521	2.0	2.3	19	1.0	1.9
Convulsions and Epilepsy	5669	1.0	1.7	191	1.0	1.8
Angina	1245	1.0	1.3	40	1.0	1.6
Ear, nose, and throat inflections	1794	1.0	1.2	56	1.0	1.4
Asthma	1986	1.0	1.4	61	1.0	1.2
Iron deficiency anaemia	477	1.0	1.9	12	0.0	0.8
Hypertension	238	1.0	1.4	5	0.0	0.4
Other Vaccine Preventable	57	1.0	2.2	<5		
Pelvic Inflammatory Disease	126	2.0	3.1	<5		
Nutritional deficiencies	<5			<5		

* rounded to the nearest 5 to avoid disclosure on the number of Nutritional Deficiencies spells.

Table A4. Adult ACSC mortality rates per 1,000 person-years, by ID status and 'main-cause of death' status, 2011-19 (sorted by decreasing main-cause SMR in ID group)

ACSC	Main-cause						All-cause					
	Crude mortality rate (CMR)				ID mortality rate age-sex standardized to 'No ID' population		Crude mortality rate (CMR)				ID mortality rate age-sex standardized to 'No ID' population	
	No ID		ID				No ID		ID			
	CMR	CMR 95% CI	CMR	CMR 95% CI	SMR	SMR 95% CI	CMR	CMR 95% CI	CMR	CMR 95% CI	SMR	SMR 95% CI
Any ACSC	1.03	(1.00, 1.06)	1.67	(1.47, 1.90)	2.62	(2.36, 2.91)	3.90	(3.84, 3.95)	5.66	(5.27, 6.08)	8.79	(8.30, 9.30)
COPD	0.80	(0.78, 0.83)	0.57	(0.46, 0.71)	1.06	(0.90, 1.25)	1.73	(1.70, 1.77)	1.16	(1.00, 1.36)	2.12	(1.88, 2.38)
Convulsions and Epilepsy	0.02	(0.02, 0.02)	0.69	(0.56, 0.85)	0.79	(0.65, 0.95)	0.10	(0.09, 0.11)	2.36	(2.11, 2.63)	2.76	(2.49, 3.06)
Asthma	0.03	(0.02, 0.03)	0.10u	(0.06, 0.17)	0.17u	(0.12, 0.26)	0.09	(0.08, 0.10)	0.23	(0.16, 0.33)	0.39	(0.30, 0.52)
Hypertension	0.03	(0.03, 0.04)	0.06u	(0.03, 0.12)	0.14u	(0.09, 0.22)	1.15	(1.12, 1.18)	0.76	(0.63, 0.93)	1.56	(1.36, 1.78)
Congestive Heart Failure	0.08	(0.07, 0.09)	0.07u	(0.04, 0.14)	0.13u	(0.09, 0.21)	1.15	(1.12, 1.18)	1.25	(1.08, 1.46)	2.18	(1.94, 2.44)
Influenza and Pneumonia	0.03	(0.02, 0.03)	0.07u	(0.04, 0.14)	0.12u	(0.07, 0.20)	0.04	(0.03, 0.04)	0.08u	(0.05, 0.15)	0.13u	(0.08, 0.20)
Cellulitis	0.02	(0.02, 0.03)	0.04u	(0.02, 0.09)	0.08u	(0.04, 0.15)	0.05	(0.04, 0.05)	0.09u	(0.05, 0.16)	0.16u	(0.10, 0.24)
Pyelonephritis	0.01	(0.00, 0.01)	0.04u	(0.02, 0.09)	0.06u	(0.03, 0.13)	0.01	(0.01, 0.01)	0.08u	(0.05, 0.15)	0.12u	(0.07, 0.20)
Dehydration and Gastroenteritis	0.01	(0.00, 0.01)					0.03	(0.03, 0.04)	0.12u	(0.07, 0.19)	0.27u	(0.20, 0.38)
Angina	0.00u	(0.00, 0.00)					0.03	(0.02, 0.03)				
Gangrene							0.02	(0.01, 0.02)	0.04u	(0.02, 0.09)	0.06u	(0.03, 0.13)
Pelvic Inflammatory Disease							0.00u	(0.00, 0.00)				
Nutritional Deficiencies							0.00u	(0.00, 0.00)				
Ear, nose and throat infections							0.00u	(0.00, 0.00)				
Dental conditions							0.00u	(0.00, 0.00)				
Iron deficiency anaemia												
Other Vaccine Preventable												
Perforated/bleeding ulcer												
Diabetes Complications												

Note: letter ‘u’ indicates that the figure may not be reliable due to the small number of cases (between 5-20).

Table A5. Statistics on hospitalization spells among adults alive on census day who had at least one unplanned hospitalisation due to ACSC during the study period, by ID status and ACSC, 2011-19 (sorted by decreasing order of 'Number of hosp. spells' in ID)

ACSC	No ID			ID			p-value on the group difference in 'Mean number of spells'*
	Number of hosp. spells	Number of hosp. spells as % of all ACSC spells	Mean number of spells (95% CI)	Number of hosp. spells	Number of hosp. spells as % of all ACSC spells	Mean number of spells (95% CI)	
Any ACSC	30692**	100	1.87 (1.85; 1.90)	924*	100	1.97 (1.84; 2.10)	<0.001
Seizures and Epilepsy	5669	18.5	2.20 (2.14; 2.26)	191	20.7	2.36 (2.04; 2.72)	<0.001
COPD	5663	18.5	2.36 (2.30; 2.42)	177	19.2	2.90 (2.49; 3.36)	<0.001
Influenza and Pneumonia	4342	14.1	1.19 (1.16; 1.23)	143	15.5	1.25 (1.06; 1.48)	<0.001
Asthma	1986	6.5	1.88 (1.80; 1.96)	61	6.6	2.10 (1.61; 2.70)	<0.001
Cellulitis	2395	7.8	1.28 (1.23; 1.33)	59	6.4	1.20 (.92; 1.55)	.001

Ear, nose and throat infections	1794	5.8	1.21 (1.16; 1.27)	56	6.1	1.40 (1.06; 1.82)	<0.001
Congestive Heart Failure	2061	6.7	1.33 (1.28; 1.39)	56	6.1	1.47 (1.11; 1.91)	<0.001
Diabetes Complications	2133	6.9	2.15 (2.06; 2.24)	42	4.5	1.40 (1.01; 1.89)	<0.001
Angina	1245	4.1	1.24 (1.18; 1.31)	40	4.3	1.05 (.75; 1.43)	<0.001
Dehydration and Gastroenteritis	1147	3.7	1.12 (1.05; 1.18)	39	4.2	1.15 (.82; 1.57)	.345
Pyelonephritis	521	1.7	1.08 (.99; 1.18)	19	2.1	1.06 (.64; 1.65)	.635
Iron deficiency anaemia	477	1.6	1.11 (1.01; 1.22)	12	1.3	1.09 (.56; 1.91)	.830
Perforated/bleeding ulcer	187	0.6	1.03 (.89; 1.19)	7	0.8	1.00 (.40; 2.06)	.854
Gangrene	351	1.1	1.14 (1.02; 1.26)	7	0.8	1.17 (.47; 2.40)	.871
Dental conditions	298	1	1.04 (.93; 1.17)	5	0.5	1.00 (.32; 2.33)	.844

Hypertension	238	0.8	1.08 (.95; 1.23)	5	0.5	1.00 (.32; 2.33)	.703
Pelvic Inflammatory Disease	126	0.4	1.17 (.97; 1.39)				
Other Vaccine Preventable	57	0.2	1.46 (1.11; 1.89)				
Nutritional deficiencies							

* T-test. Normality was evaluated through a visual inspection of the histogram.

** 1.5% of all unplanned ACSC hospitalisation spells had more than one ACSC as a cause. For analytical purposes, the columns ‘Number of hosp. spells’ treat such spells as separate spells with their own one cause rather than one spell. The number of spells in this table is therefore higher than the true number that could be calculated from Table A1.

Note: Columns ‘Mean number of spells’ refer to the number of spells counted for each person who had one or more unplanned hospitalisation for a given ACSC.

Table A6. Number and percent of adults alive on census day who died during the study period and whose death certificate mentions ACSC as a cause of death (any diagnostic position), by ID status, 2011-19 (sorted by decreasing order of ID n)

ACSC	No ID			ID			Stat. sig. (p-value) on group difference in 'Percent of baseline population'*
	n	Percent of baseline population (95% CI)	Percent of deaths involving ACSC	n	Percent of baseline population (95% CI)	Percent of deaths involving ACSC	
Any ACSC	18246	3.22 (3.18;	100.0	763	4.53 (4.22;	100.0	<0.001
Convulsions and Epilepsy	482	.09 (.08; .09)	2.6	318	1.89 (1.69;	41.7	<0.001
Congestive Heart Failure	5373	.95 (.92; .97)	29.4	169	1.00 (.86;	22.1	.473
COPD	8119	1.43 (1.40;	44.5	157	.93 (.79; 1.09)	20.6	<0.001
Hypertension	5365	.95 (.92; .97)	29.4	103	.61 (.50; .74)	13.5	<0.001
Asthma	404	.07 (.06; .08)	2.2	31	.18 (.13; .26)	4.1	<0.001
Dehydration and Gastroenteritis	146	.03 (.02; .03)	0.8	16	.10 (.05; .15)	2.1	<0.001
Cellulitis	223	.04 (.03; .04)	1.2	12	.07 (.04; .12)	1.6	.042
Influenza and Pneumonia	164	.03 (.02; .03)	0.9	11	.07 (.03; .12)	1.4	.007
Pyelonephritis	53	.01 (.01; .01)	0.3	11	.07 (.03; .12)	1.4	<0.001
Gangrene	78	.01 (.01; .02)	0.4	5	.03 (.01; .07)	0.7	.088
Angina	138	.02 (.02; .03)	0.8	<5			
Pelvic Inflammatory Disease	11	.00 (.00; .00)	0.1	<5			
Nutritional Deficiencies	11	.00 (.00; .00)	0.1	<5			
Ear, nose and throat infections	7	.00 (.00; .00)	0.0	<5			
Dental conditions	8	.00 (.00; .00)	0.0	<5			
Diabetes Complications	<5			<5			
Iron deficiency anaemia	<5			<5			
Perforated/bleeding ulcer	<5			<5			
Other Vaccine Preventable	<5			<5			

* z-test for proportions using the 'prtesti' function in Stata 17.

Table A7. Adult ACSC mortality ratios, cases with ACSC mentioned as a cause of death in any diagnostic position on the death certificate, ID cohort (numerator) compared with No ID cohort (denominator), 2011-19 (sorted by decreasing SMR)

ACSC	Mortality Ratio (MR)	MR 95% CI	Standardized Mortality Ratio (SMR)	SMR 95% CI
Any ACSC	1.45	(1.35, 1.56)	2.26	(2.08, 2.44)
Convulsions and Epilepsy	22.93	(19.84, 26.47)	26.83	(23.11, 31.15)
Pyelonephritis	7.21u	(3.40, 13.97)	10.70u	(5.46, 20.99)
Dehydration and Gastroenteritis	3.81u	(2.12, 6.40)	8.73u	(5.06, 15.04)
Asthma	2.67	(1.79, 3.85)	4.57	(3.07, 6.81)
Gangrene	2.23u	(0.70, 5.42)	3.88u	(1.45, 10.42)
Influenza and Pneumonia	2.33u	(1.14, 4.28)	3.58u	(1.80, 7.12)
Cellulitis	1.87u	(0.95, 3.33)	3.32u	(1.77, 6.20)
Congestive Heart Failure	1.09	(0.93, 1.27)	1.90	(1.60, 2.25)
Hypertension	0.67	(0.54, 0.81)	1.36	(1.10, 1.68)
COPD	0.67	(0.57, 0.79)	1.22	(1.03, 1.45)
Other Vaccine Preventable				

Pelvic Inflammatory Disease				
Angina				
Iron deficiency anaemia				
Perforated/bleeding ulcer				
Diabetes Complications				
Dental conditions				
Ear, nose and throat infections				
Nutritional Deficiencies				

Note: letter 'u' indicates that the figure may not be reliable due to the small number of cases (between 5-20).
