# Supplementary material for: Hospitalisations and deaths due to ambulatory care sensitive conditions among adults with and without intellectual disabilities in Scotland: a cohort study
Source: BMJ Open. 2026 Mar 2;16(3):e105055. doi: 10.1136/bmjopen-2025-105055 (PMC12958873; doi:10.1136/bmjopen-2025-105055)
Supplement: Supplementary file 3 [file bmjopen-2025-105055.reviewer_comments.pdf]

PEER REVIEW HISTORY

BMJ Open publishes all reviews undertaken for accepted manuscripts. Reviewers are asked to complete a checklist review form and are provided with free text boxes to elaborate on their assessment. These free text comments are reproduced below.

ARTICLE DETAILS

Title (Provisional)

Hospitalisations and deaths due to Ambulatory Care Sensitive Conditions among adults with and without Intellectual Disabilities in Scotland: a cohort study

Authors

Sosenko, Filip; Cairns, Deborah; Jani, Bhautesh; Ward, Laura McKernan; Truesdale, Maria; Hughes-McCormack, Laura; Henderson, Angela; Melville, Craig

---

VERSION 1 - REVIEW

**Reviewer**	**1**
**Name**	**Gangadharan, Satheesh**
**Affiliation**	**Loughborough University Loughborough School of Design and Creative Arts, Design school**
**Date**	**26-May-2025**
**COI**	**None**

This study is focused on an important issue and health inequality of people with ID is an important one.

The study using data linkage between census data, hospital admission and death data enabled a reasonably long period of follow up although there is some limitation related to sample size leading wide confidence intervell. This has been mentioned specifically as a weakness.

The focus on ACSC related hospitalisation and death related ACSC and specifically use of standardised Incidence ration for these to account for the effect of population prevalence is the particular strength of the study.

Study is well described and succint with apropriate references. I have the following questions / suggestions (I would consider these in "minor review category).

1. I see that on the ID side people with additional diagnosis of autism was included but on the general population side autistic people without autism were excluded. I can understand that this could be due to the potential confounding that this could bring. At the same time, I

am not sure why autism was spelled out as opposed other neurodevelopmental or mental health conditions like ADHD, severe mental illness etc.

2. PPI- says that "people first" a group of expert by experience were in the steering committee- would authors comment on how they were able to influence the study.

3. abbreviation "SIR" has been used extensively but I can see any places where the full form of this has been described.

**Reviewer**	**2**
**Name**	**Durbin, Janet**
**Affiliation**	**Center for Addiction and Mental Health**
**Date**	**30-Jun-2025**
**COI**	**None**

This cohort study compared rates of hospitalization and death for Ambulatory Care Sensitive Conditions (ACSCs) among adults with and without intellectual and developmental disabilities in Scotland. Using linked administrative data, outcomes over a 10 year period ending in 2019 were assessed. A study rationale is that poorer primary care (PC) can contribute to higher rates of ACSC admissions, so findings in part can inform understanding of quality of PC for persons with IDD. This is important as persons with IDD have more medical complexity and shorter life expectancy than those without IDD. Use of population administrative data and methodological rigor are study strengths. However several areas need improvement.

General: To make paper easier to follow, try to be clear for each component on what data are being reported (e.g., unadjusted, adjusted (for age, sex, prevalence, main cause ACSC, all cause ACSC), crude rates, ratio rates). There is a lot for the reader to follow re what each analysis includes and how it differs from the previous.

Controlling for prevalence: The study introduction notes this as a common gap in prior studies and a strength of the present study. However this value-add needs to be clearly threaded throughout. This starts with the abstract where prevalence should be added to the aim and method. In the paper it should be added in the aim paragraph, as a section in the methodology, and more clearly presented in the results section. How Table 4 prevalence results relate to SIR results was unclear to me and I found the page 15 paragraph starting with ‘taking prevalence into consideration….’ confusing. SIR results could be added to table 4 and clearly interpreted in the text.

Definitions: Study measures and terms need to be defined clearly and where relevant a rationale for use provided (e.g., SIR, spell).

Age and sex gender results (page 14): This analysis is important. It could use a subtitle to stand out more and would be easier to follow if reported in a table. The age cutoff moves between 55 and 65. Can this be explained?

Discussion and implications: A main finding is that, with prevalence adjustment, ACSC admissions are actually lower for individuals with IDD, raising the possibility of persons with IDD receiving superior PC. While some caveats are offered, this finding could use more discussion. Lower hospitalization may not mean better care, especially with seizures, epilepsy and COPD being so prevalent and causes of mortality within a younger cohort. Diagnostic overshadowing is mentioned. Can the authors offer any other insights? Also what further research would be helpful? For example, how many of the ACSC admissions have had a recent PC visit? The paper does appropriately note near the end a need for more attention by PC to epilepsy, seizures and COPD.

**Reviewer**	**3**
**Name**	**Kelson, Zoe**
**Affiliation**	**University of Exeter, Mathematics**
**Date**	**20-Aug-2025**
**COI**	**None**

This study aims to explore how well the primary care system in Scotland works for adults with Intellectual Disabilities (ID), using the rate of unplanned hospital admissions for Ambulatory Care Sensitive Conditions (ACSC) as a proxy indicator.

Reviewer comments:

"crude and age-sex adjusted incidence rates and ratios; cumulative incidence"

Can statistical methods please be summarised in the Abstract?

"Adults with ID under the age of 55 had only a slightly higher risk of an unplanned ACSC hospitalisation than their general population counterparts. After adjusting for different ACSC prevalence in ID and non-ID cohorts this difference in risk disappeared. These findings contrast with existing evidence from England, where a much higher risk of unplanned ACSC hospitalisations was found among people with ID. Adults with ID had a higher risk of dying due to ACSC than adults without ID. Conclusions"

Can statistical outcomes please be reported alongside study findings in the Abstract?

"A population-based retrospective cohort data linkage study of adult respondents to Scotland’s 2011 Census." [Abstract]

and

"The research was a population-based cohort data linkage study of people who took part in Scotland’s 2011 Census and were at least 18 years old on census day (27 March 2011)."

Can the census survey design and sample weighting methods please be described in more detail?

"Self- or proxy-reported ID status from the Census was linked to hospital admissions data and deaths data. " [Abstract]

and

"The individual’s ID status was determined from census data; one of the questions regarded disabilities and the list included ‘learning disability’ (the term much more widely used in the UK than ‘intellectual disability’, but with the same meaning). Census information was provided by a proxy where the respondent was not capable of completing the form. People who self- or proxy-identified as from the general population but who subsequently died of ID (ICD-10 codes F70, F71, F72, F73, F78, F79, F84) were excluded (n=30)."

Can the authors please acknowledge the potential for self-reporting and recall bias?

"The cohort was followed until the end of 2019. "

Can the authors please comment on whether the study outcomes are still relevant today?

Might patterns have changed since 2011-2019?

"People aged 18+ on Census day (27 March 2011), including all adults with ID (n=16,840) and a 15% randomly selected comparator sample of adults without ID (n=566,074)"

Can the authors please demonstrate whether the 'no ID' cohort is comparable to the 'ID' cohort?

Did the authors consider matching the cohorts on potential confounders?

Can the authors please provide their rationale for sampling 15% of the no ID population (i.e. why not more or less than this)? Was a sample size calculation conducted?

"The three datasets used for this project (Scotland’s 2011 Census, hospital admissions, death certificate data) were provisioned by Electronic Data Research and Innovation Service (eDRIS, part of Public Health Scotland) via the Scottish National Safe Haven, a platform for researchers working with large databases of electronic health records"

and

"We will use complete case analysis. However, we may include dummy variables for categories that are missing if the impact of missing data could restrict analysis. Any errors or ambiguous deaths will be listed as an unknown cause. "

Can the authors please confirm if there was any missing data?

How much missing data was there, and can it be considered to be missing at random?

"The prevalence of specific ACSC among the ID population in Scotland is unknown, and we did not have access to primary care data from which it could be estimated. We therefore used data from England, specifically the National Health Service (NHS) data series 'Health and Care of People with Learning Disabilities'iii. We regard the Scottish and English ID populations to be close enough in character for it to not be an issue for our analysis. While only six of 19 ACSC conditions are covered by this data source, collectively they are responsible for over half of all unplanned ACSC hospitalisations in Scotland (see below). "

Can the authors please conduct a sensitivity analysis to explore uncertainty in this data?

"The analysis focused on incidence rate ratios, both crude and age-sex adjusted."

Can the authors please specify how they computed age-sex adjusted IR ratios?

"In all analyses statistical significance was defined at the conventional 5% level and determined by examining the overlap of 95% Confidence Intervals."

Can the authors please clarify the methodology applied for generating confidence intervals?

Furthermore, noting that "letter 'u' indicates that the figure may not be reliable due to the small number of cases (between 5-20)", can the authors please clarify how they attempted to account for small numbers in their methodology for computing IRRs and CIs?

Can the authors please also conduct statistical tests in support of study inferences?

Can the authors please conduct multivariate analyses to adjust for potential confounding?

"Table 1. Age, sex and area deprivation profile of adults on Census Day, by ID status"

Can more detail please be provided in this table, indicating the numbers in each comparable cohort of 'No ID' and 'ID' and defining 'SIMD' in the footnote?

Can more demographics and characteristics of each cohort please be included in this table?

"Table 2. Statistics on adults alive on census day who had at least one unplanned hospitalisation due to ACSC during the study period, by ID status and ACSC, 2011-19 (sorted by decreasing order of 'Number of hosp. spells' in ID) "

and

"Table 5. Number and percent of adults alive on census day who died during the study period and whose death certificate mentions ACSC as 'main-cause of death' or 'all-cause of death', by ID status, 2011-19 (sorted by decreasing order of ID main-cause n)"

Can confidence intervals and p-values for statistical comparisons please also be reported in these tables?

Can the authors please ensure that all abbreviations are defined at first use throughout the article (for example, SIR and CMR)?

"Key limitation: small absolute size of the ID population in Scotland entailed wide confidence intervals in most cases" [Strengths and Limitations of this study]

and

"The study's biggest limitation was the small size of the ID population in Scotland. This meant that our confidence intervals were often too wide to draw any conclusions. When confidence intervals are wide it is not possible to know whether differences in rates and ratios reflect the reality or are just due to random chance. Another limitation was that we had prevalence data only for six out of 19 ACSCs. However, within those six, two (convulsions and epilepsy, COPD) are the leading causes of unplanned ACSC hospitalisations in ID and four are the leading causes of all-cause mortality. Therefore, this limitation is relatively minor. Also related to the data on prevalence, it is a limitation that we did not have a joint, individual-level source of information on hospitalisations and prevalence. Had we had such joint data, it could turn out that the prevalence of different ACSCs is in fact higher than reported in Table 4. "

A discussion on the study limitations has been provided.

Thanks for providing a copy of the STROBE statement.

VERSION 1 - AUTHOR RESPONSE

Reviewer: 1
Prof. Satheesh Gangadharan, Loughborough University Loughborough School of Design and Creative Arts, Leicestershire Partnership NHS Trust

	Authors' response
1. I see that on the ID side people with additional diagnosis of autism was included but on the general population side autistic people without autism were excluded. I can understand that this could be due to the potential confounding that this could bring. At the same time, I am not sure why autism was spelled out as opposed other neurodevelopmental or mental health conditions like ADHD, severe mental illness etc.	Yes, it was to do with potential confounding. We agree that controlling for ADHD or severe mental illness would have been beneficial, but unfortunately we did not have data on these.
2. PPI- says that "people first" a group of expert by experience were in the steering committee- would authors comment on how they were able to influence the study.	Since 2015, when the Scottish Government funded the establishment of the Scottish Learning Disabilities Observatory, the steering group has included a range of experts, including experts by experience from People First. From the outset, these members have played a central role in shaping the Observatory's research priorities and

	ensuring that our work remains grounded in lived experience.<br><br>Steering group meetings have always focused strongly on discussing findings, identifying future directions, and ensuring that dissemination activities are accessible and impactful. During the initial phase of the Observatory's work, early findings were presented to the group, and discussions with experts by experience helped identify priority areas for further research. These conversations highlighted the importance of tackling health care inequalities, particularly within primary care.<br><br>Insights from experts by experience were supported by emerging evidence from our first phase of research, which showed that people with intellectual disabilities experience significant inequalities in primary care and higher rates of potentially avoidable hospital admissions. Experts by experience also shared their own barriers to accessing health care through lived experience, including through collaborative projects such as co-produced videos on barriers and facilitators to primary care access, which were widely disseminated to raise awareness.<br><br>As a result of these discussions and collaborations, primary health care became a core research theme for subsequent phases of the Observatory's work, and the research question for the current study was directly informed by the priorities identified by experts by experience.
3. abbreviation "SIR" has been used extensively but I can see any places where the full form of this has been described.	Full form added just before Table 3.

Reviewer: 2

Dr. Janet Durbin, Center for Addiction and Mental Health

	Authors' response
General: To make paper easier to follow, try to be clear for each component on what data are being reported (e.g., unadjusted, adjusted (for age, sex, prevalence, main cause ACSC, all cause ACSC), crude rates, ratio rates). There is a lot for the reader to follow re what each analysis includes and how it differs from the previous.	Clarified throughout the text.
Controlling for prevalence: The study introduction notes this as a common gap in prior studies and a strength of the present study. However this value-add needs to be clearly threaded throughout. This starts with the abstract where prevalence should be added to the aim and method. In the paper it should be added in the aim paragraph, as a section in the methodology, and more clearly presented in the results section. How Table 4 prevalence results relate to SIR results was unclear to me and I found the page 15 paragraph starting with 'taking prevalence into consideration....' confusing. SIR results could be added to table 4 and clearly interpreted in the text.	Text added throughout, and a new table with SIR results has also been added for clarity (now Table 4).
Definitions: Study measures and terms need to be defined clearly and where relevant a rationale for use provided (e.g., SIR, spell).	Added.
Age and sex gender results (page 14): This analysis is important. It could use a subtitle to stand out more and would be easier to follow if reported in a table. The age cutoff moves between 55 and 65. Can this be explained?	Table added. Cutoffs explained in a note under the table.
Discussion and implications: A main finding is that, with prevalence adjustment, ACSC admissions are actually lower for individuals with IDD, raising the possibility of persons with IDD receiving superior PC. While some caveats are offered, this finding could use more	Two paragraphs added to the discussion.

discussion. Lower hospitalization may not mean better care, especially with seizures, epilepsy and COPD being so prevalent and causes of mortality within a younger cohort. Diagnostic overshadowing is mentioned. Can the authors offer any other insights? Also what further research would be helpful? For example, how many of the ACSC admissions have had a recent PC visit? The paper does appropriately note near the end a need for more attention by PC to epilepsy, seizures and COPD.	

Reviewer: 3

Prof. Zoe Kelson, University of Exeter

	Authors' response
"crude and age-sex adjusted incidence rates and ratios; cumulative incidence" Can statistical methods please be summarised in the Abstract?	Added.
"Adults with ID under the age of 55 had only a slightly higher risk of an unplanned ACSC hospitalisation than their general population counterparts. After adjusting for different ACSC prevalence in ID and non-ID cohorts this difference in risk disappeared. These findings contrast with existing evidence from England, where a much higher risk of unplanned ACSC hospitalisations was found among people with ID. Adults with ID had a higher risk of dying due to ACSC than adults without ID. Conclusions"<br>Can statistical outcomes please be reported alongside study findings in the Abstract?	Added.
"A population-based retrospective cohort data linkage study of adult respondents to Scotland's 2011 Census." [Abstract] and<br>"The research was a population-based cohort data linkage study of people who took part in Scotland's 2011 Census and	Added before the sentence starting 'The study was approved by Scotland's Statistics Public Benefit and Privacy Panel…'.

were at least 18 years old on census day (27 March 2011)."<br>Can the census survey design and sample weighting methods please be described in more detail?	
"Self- or proxy-reported ID status from the Census was linked to hospital admissions data and deaths data. " [Abstract]<br>and<br>"The individual's ID status was determined from census data; one of the questions regarded disabilities and the list included 'learning disability' (the term much more widely used in the UK than 'intellectual disability', but with the same meaning). Census information was provided by a proxy where the respondent was not capable of completing the form. People who self- or proxy-identified as from the general population but who subsequently died of ID (ICD-10 codes F70, F71, F72, F73, F78, F79, F84) were excluded (n=30)."<br>Can the authors please acknowledge the potential for self-reporting and recall bias?	Point about self-reporting added to limitations. Recall bias is not an issue in the case of intellectual disabilities.
"The cohort was followed until the end of 2019. "<br>Can the authors please comment on whether the study outcomes are still relevant today? Might patterns have changed since 2011-2019?	Included in the new section on future research.
"People aged 18+ on Census day (27 March 2011), including all adults with ID (n=16,840) and a 15% randomly selected comparator sample of adults without ID (n=566,074)"<br>Can the authors please demonstrate whether the 'no ID' cohort is comparable to the 'ID' cohort?<br>Did the authors consider matching the cohorts on potential confounders?	Sorry, we are not sure what the Reviewer means by 'ID and non-ID cohorts being (or not) comparable'.<br><br>Matching on confounders should only be used if data collection from subjects is expensive, to maximize the amount of information obtained per subject (Rothman 2012; Rothman, Greenland, and Lash 2008). In our retrospective cohort study (as in the vast majority of retrospective cohort studies) all of the data was available to researchers, so there was no dilemma 'should we spend

	more money on getting more subjects, and which ones'. Matching in cohort studies could decrease the study's statistical power (Greenland and Morgenstern 1990) (Faresjö and Faresjö 2010). It also prohibits investigation of the effect of matching variables on the outcome and makes it impossible to study interactions (how the effect of the exposure variable is modified by the levels of the matching variable) (Szklo and Nieto 2019).
Can the authors please provide their rationale for sampling 15% of the no ID population (i.e. why not more or less than this)? Was a sample size calculation conducted?	Added a reference to power calculations.
"The three datasets used for this project (Scotland's 2011 Census, hospital admissions, death certificate data) were provisioned by Electronic Data Research and Innovation Service (eDRIS, part of Public Health Scotland) via the Scottish National Safe Haven, a platform for researchers working with large databases of electronic health records"<br>and<br>"We will use complete case analysis. However, we may include dummy variables for categories that are missing if the impact of missing data could restrict analysis. Any errors or ambiguous deaths will be listed as an unknown cause. "<br>Can the authors please confirm if there was any missing data?<br>How much missing data was there, and can it be considered to be missing at random?	Information about missingness added to the 'Study Design' section.<br>We are not sure where the Reviewer sourced this text: "We will use complete case analysis. However, we may include dummy variables for categories that are missing if the impact of missing data could restrict analysis. Any errors or ambiguous deaths will be listed as an unknown cause." This text is not in the paper.
"The prevalence of specific ACSC among the ID population in Scotland is unknown, and we did not have access to primary care data from which it could be estimated. We therefore used data from England, specifically the National Health Service (NHS) data series 'Health and Care of People with Learning Disabilities'iii. We regard the Scottish	A sensitivity analysis was not possible. We have added this to study limitations.

and English ID populations to be close enough in character for it to not be an issue for our analysis. While only six of 19 ACSC conditions are covered by this data source, collectively they are responsible for over half of all unplanned ACSC hospitalisations in Scotland (see below).<br>"<br>Can the authors please conduct a sensitivity analysis to explore uncertainty in this data?	
"The analysis focused on incidence rate ratios, both crude and age-sex adjusted."<br>Can the authors please specify how they computed age-sex adjusted IR ratios?	Added to the 'Data Analysis' section.
"In all analyses statistical significance was defined at the conventional 5% level and determined by examining the overlap of 95% Confidence Intervals."<br>Can the authors please clarify the methodology applied for generating confidence intervals?	Added to the 'Data Analysis' section.
Can the authors please also conduct statistical tests in support of study inferences?	Results of statistical tests have been added to Table 2, Table A5, Table 6 and Table A6. Note that the editorial team have requested that the original Tables 2, 6 and 7 be split into separate tables due to the excessive number of columns.
Can the authors please conduct multivariate analyses to adjust for potential confounding?	The two most important confounders - age and sex – have been adjusted for through age-sex standardization of rates and ratios. Lack of ethnicity analysis has been added to study limitations and explained.<br>Adjustment for economic position (proxied by SIMD) was purposefully not done; poverty is both an outcome of ID as well as its determinant, and outcomes of the exposure (other than the outcome of interest, here hospitalisations) should not be included in the causal path, as they may bias the effect of the exposure (Pearl 2014) (Greenland 2003).
"Table 1. Age, sex and area deprivation profile of adults on Census Day, by ID status"<br>Can more detail please be provided in	Added.

this table, indicating the numbers in each comparable cohort of 'No ID' and 'ID' and defining 'SIMD' in the footnote?	
Can more demographics and characteristics of each cohort please be included in this table?	Unfortunately, those three variables are the only ones that were provided to us by eDRIS.
"Table 2. Statistics on adults alive on census day who had at least one unplanned hospitalisation due to ACSC during the study period, by ID status and ACSC, 2011-19 (sorted by decreasing order of 'Number of hosp. spells' in ID) " and "Table 5. Number and percent of adults alive on census day who died during the study period and whose death certificate mentions ACSC as 'main-cause of death' or 'all-cause of death', by ID status, 2011-19 (sorted by decreasing order of ID main-cause n)" Can confidence intervals and p-values for statistical comparisons please also be reported in these tables?	Added.
Can the authors please ensure that all abbreviations are defined at first use throughout the article (for example, SIR and CMR)?	Done.

VERSION 2 - REVIEW

**Reviewer**	**2**
**Name**	**Durbin, Janet**
**Affiliation**	**Center for Addiction and Mental Health**
**Date**	**01-Dec-2025**
**COI**	

The manuscript is much improved. Thank you.

Please define 'spell' in the text (it is defined in the appendix but would be easier for reader and simple to define in the main paper.

Please define IRR and SIR in the data analysis section. These are sort of referenced but not explicitly defined.

I could not see supplement tables A2, A3...

**Reviewer**	**3**
**Name**	**Kelson, Zoe**
**Affiliation**	**University of Exeter, Mathematics**
**Date**	**06-Nov-2025**
**COI**	

Thanks to the authors for the amendments.

Of note, detailed responses to reviewer specifying the changes made and a copy of the article clearly showing tracked changes would help with the re-review process.

Author response:

"Added a reference to power calculations."

Article text:

"The two study cohorts consisted of all adults with ID (n=16,840) and a 15% randomly selected comparator sample from the general population (n=566,074). (See [36] for more details, including power calculations). "

Reviewer request:

Can the rationale for a 15% sample please be expanded on within the article?

Author response:

"Information about missingness added to the 'Study Design' section"

Article text:

"Data for 7% of the population who did not return the census form were imputed by the Census team. (As such, no weighting scheme is supposed to be applied in the analysis of census records). However, our study excluded those imputed records, as there was too much uncertainty that someone with imputed ID status really had ID. Where the census form was returned with missing key demographic characteristics (age, sex, etc.), we could not evaluate the extent of missingness / imputation, as the NRS did not publish that information. The area deprivation variable provided by the NRS was not subject to imputation and had a large proportion of missing values (Table 1)."

Reviewer requests:

Can the authors please conduct a sensitivity analysis including imputed records?

Can the authors please specify the proportions of missing data?

Can missing data, and whether it can be considered to be missing at random, please be further discussed in the study limitations?

Reviewer previous comments:

Can the authors please specify how they computed age-sex adjusted IR ratios?

and

Can the authors please clarify the methodology applied for generating confidence intervals?

Author response:

"Added to the 'Data Analysis' section."

Article text:

"The analysis focused on incidence rate ratios, both crude and age-sex standardized. The reference population for age-sex adjustment was people without ID in the analysis dataset. Rates, rate ratios and 95% CIs were calculated using the 'stir' command in Stata 17....

In all analyses statistical significance was defined at the conventional 5% level. Differences in group means and proportions were investigated via t-test, except for analyses that adjusted for ACSC prevalence; we examined the overlap of 95% Confidence Intervals (CI) there instead."

Reviewer requests:

Can the authors please further specify the equations and formula applied for the computation of ratios and confidence intervals in the 'Data Analyses' section?

Can the authors please clarify how they assessed normality for the choice of applying the parametric t-test?

Author response:

"Results of statistical tests have been added to Table 2, Table A5, Table 6 and Table A6."

Reviewer request:

Can the methods applied in these tables please also be specified in associated footnotes?

Author response:

"The two most important confounders - age and sex – have been adjusted for through age-sex standardization of rates and ratios. Lack of ethnicity analysis has been added to study limitations and explained. Adjustment for economic position (proxied by SIMD) was purposefully not done; poverty is both an outcome of ID as well as its determinant, and outcomes of the exposure (other than the outcome of interest, here hospitalisations) should not be included in the causal path, as they may bias the effect of the exposure (Pearl 2014) (Greenland 2003)."

Reviewer request:

Can the authors please further incorporate this response to reviewer into the article?

Author response:

"Unfortunately, those three variables are the only ones that were provided to us by eDRIS"

Reviewer request:

Can the authors please acknowlewdge this in the discussion of the study limitations?

VERSION 2 - AUTHOR RESPONSE

Reviewer: 2
Dr. Janet Durbin, Center for Addiction and Mental Health

Please define 'spell' in the text (it is defined in the appendix but would be easier for reader and simple to define in the main paper.	Definition added on first use of 'spell'.
Please define IRR and SIR in the data analysis section. These are sort of referenced but not explicitly defined.	Definitions added to the Data Analysis section.
I could not see supplement tables A2, A3…	They are in the Appendix. (Not in Supplemenatry File).

Reviewer: 3
Prof. Zoe Kelson, University of Exeter

Can the rationale for a 15% sample please be expanded on within the article?	Added.
Can the authors please conduct a sensitivity analysis including imputed records?	This is not possible as we did not ask the NRS for this 7% of imputed cases at all, knowing that we would not be using them.
Can the authors please specify the proportions of missing data?	This is not possible as the NRS have not published information on item missingness and the extent of imputation. We have added an explanation to the Methods section why this is not possible.
Can missing data, and whether it can be considered to be missing at random, please be further discussed in the study limitations?	A paragraph added to Study Limitations.

Can the authors please further specify the equations and formula applied for the computation of ratios and confidence intervals in the 'Data Analyses' section?	Formula for the computation of ratios and confidence intervals added to the Data Analysis section.
Can the authors please clarify how they assessed normality for the choice of applying the parametric t-test?	Added to the Methods section.
Can the methods applied in Table 2, Table A5, Table 6 and Table A6 please also be specified in associated footnotes?	Added.
"The two most important confounders - age and sex – have been adjusted for through age-sex standardization of rates and ratios. Lack of ethnicity analysis has been added to study limitations and explained. Adjustment for economic position (proxied by SIMD) was purposefully not done; poverty is both an outcome of ID as well as its determinant, and outcomes of the exposure (other than the outcome of interest, here hospitalisations) should not be included in the causal path, as they may bias the effect of the exposure (Pearl 2014) (Greenland 2003)."<br>Reviewer request:<br>Can the authors please further incorporate this response to reviewer into the article?	Added to the Data Analysis section.
"Unfortunately, those three variables are the only ones that were provided to us by eDRIS"<br>Reviewer request:<br>Can the authors please acknowlewdge this in the discussion of the study limitations?	Added to study limitations.

VERSION 3 - REVIEW

Reviewer 3

Name Kelson, Zoe

Affiliation University of Exeter, Mathematics

Date 08-Jan-2026

COI

Thanks to the authors for their satisfactory responses and revisions, which have improved the reporting of this study.
